# Molecular Weight Tuning of Organic Semiconductors for Curved Organic–Inorganic Hybrid X‐Ray Detectors

**DOI:** 10.1002/advs.202101746

**Published:** 2021-11-10

**Authors:** M. Prabodhi A. Nanayakkara, Mateus G. Masteghin, Laura Basiricò, Ilaria Fratelli, Andrea Ciavatti, Rachel C. Kilbride, Sandra Jenatsch, Thomas Webb, Filipe Richheimer, Sebastian Wood, Fernando A. Castro, Andrew J. Parnell, Beatrice Fraboni, K. D. G. Imalka Jayawardena, S. Ravi P. Silva

**Affiliations:** ^1^ Advanced Technology Institute Department of Electrical and Electronic Engineering University of Surrey Guildford Surrey GU2 7XH UK; ^2^ Department of Physics and Astronomy University of Bologna Viale Berti Pichat 6/2 Bologna 40127 Italy; ^3^ National Institute for Nuclear Physics INFN section of Bologna Bologna 40127 Italy; ^4^ Department of Physics and Astronomy University of Sheffield Hicks Building Sheffield S3 7RH UK; ^5^ FLUXiM AG Katharina‐Sulzer‐Platz 2 Winterthur 8400 Switzerland; ^6^ National Physical Laboratory Teddington Middlesex TW11 0LW UK

**Keywords:** flexible substrates, molecular weight, organic electronics, photonics, radiation detectors

## Abstract

Curved X‐ray detectors have the potential to revolutionize diverse sectors due to benefits such as reduced image distortion and vignetting compared to their planar counterparts. While the use of inorganic semiconductors for curved detectors are restricted by their brittle nature, organic–inorganic hybrid semiconductors which incorporated bismuth oxide nanoparticles in an organic bulk heterojunction consisting of poly(3‐hexylthiophene‐2,5‐diyl) (P3HT) and [6,6]‐phenyl C71 butyric acid methyl ester (PC_70_BM) are considered to be more promising in this regard. However, the influence of the P3HT molecular weight on the mechanical stability of curved, thick X‐ray detectors remains less well understood. Herein, high P3HT molecular weights (>40 kDa) are identified to allow increased intermolecular bonding and chain entanglements, resulting in X‐ray detectors that can be curved to a radius as low as 1.3 mm with low deviation in X‐ray response under 100 repeated bending cycles while maintaining an industry‐standard dark current of <1 pA mm^−2^ and a sensitivity of ≈ 0.17 *μ*C Gy^−1^ cm^−2^. This study identifies a crucial missing link in the development of curved detectors, namely the importance of the molecular weight of the polymer semiconductors used.

## Graphical abstract

Solution processable organic–inorganic hybrid semiconductors for flexible curved X‐ray detector components have the potential to revolutionize diverse sectors in terms of its cost and performance. This work introduces a strategy for realizing the optimum balance for detector performance at low operating voltages with mechanical flexibility by tuning the organic semiconductor molecular weight for such curved hybrid detectors.

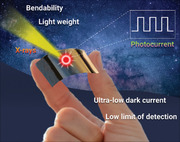

## 1 Introduction

For nearly three decades, digital imaging systems for both visible and X‐ray imaging have relied on flat imaging detectors. From an imaging standpoint, curved imaging detectors (compared to flat imaging detectors) offer several key advantages including minimization of image distortion toward the edge of the viewing field and reduced vignetting.^[^^]^ In this regard, the human eye with its curved design is still regarded as the ideal example of a curved image detector system that minimizes such effects.^[^ ^2^ ^]^ Although there is now a growing interest in developing curved image detectors,^[^ ^3^, ^4^ ^]^ mainly for imaging in the visible range of the electromagnetic spectrum, the curvatures achievable at present are restricted. This is primarily due to the underlying inorganic (mainly silicon based) semiconductor technology, which is stiff and brittle in nature and undergoes catastrophic failure upon bending. Although work reported in the literature^[^ ^5^, ^6^, ^7^ ^]^ suggests that such curved electronic device concepts are indeed possible using inorganic semiconductors, these often require complex processing that is not so easily transferred to large area imaging systems. Furthermore, these devices also suffer from poor performance due to the thinning of the semiconductor layer to ensure mechanical flexibility.^[^ ^8^, ^9^ ^]^

Among the number of potential applications for curved detectors, their use for X‐ray radiation detection in medical imaging (mammography, computed tomography etc.), clinical radiotherapy dosimetry, industrial inspection, security, and cultural heritage preservation stands out.^[^ ^10^ ^]^ In most of these applications, the “object” under evaluation consists of intricate surfaces with complex geometries, making non‐planar imaging configurations preferable. While X‐ray film provides an advantage in terms of its ability to be curved to desired shapes, the related processing times compared to digital imaging systems makes this less attractive, especially where rapid throughput is desired (e.g., in medical emergencies, manufacturing environments, and baggage scanning in airports). Recently, van Breemen et al.^[^ ^11^ ^]^ reported the development of a curved X‐ray detector based on the coupling of a commercial flexible scintillator to an organic photodiode array processed on a flexible substrate. While this study clearly demonstrates the benefits of such a technology, its performance remains limited due to the multi‐stage conversion process involved from the initial X‐ray attenuation to the signal generation process compared to direct conversion X‐ray detectors. In terms of potential material candidates available to be utilized for curved direct conversion X‐ray detectors, organic–inorganic hybrid semiconductors consisting of X‐ray attenuating nanomaterials integrated into a charge transporting organic semiconductor matrix is perhaps the most attractive.^[^ ^12^ ^]^ This technology has shown rapid advancement and can be conveniently fabricated on flexible substrates using low cost, low temperature, solution phase deposition techniques.^[^ ^10^ ^]^ In particular, the organic–inorganic hybrid detector concept which incorporated highly attenuating bismuth oxide (Bi_2_O_3_) nanoparticles (NPs) in an organic bulk heterojunction (BHJ) consisting of the p‐type polymer poly(3‐hexylthiophene‐2,5‐diyl) (P3HT) and the n‐type [6,6]‐phenyl C71 butyric acid methyl ester (PC_70_BM) is a promising architecture providing additional advantages such as high sensitivity over a broad energy range (10 kV – 15 MV) and low voltage operation.^[^ ^12^ ^]^ Preliminary studies^[^ ^12^ ^]^ for this system demonstrated its suitability as a flexible detector where detectors were deformed to a reasonable radius of curvature of ≈ 3 mm with recent developments indicating its use as a conformable detector for dose mapping in radiotherapy.^[^ ^13^ ^]^ However, in both of the above reports, the detectors suffered from high dark currents that are 3 – 4 orders of magnitude higher than state‐of‐the‐art detector technologies.^[^ ^12^, ^13^ ^]^ Recently, we reported^[^ ^14^ ^]^ a methodology to achieve ultra‐low dark currents that are well below the industrial requirements of 10 pA mm^−2^, in combination with exceptionally high sensitivities under clinical 6 MV hard X‐ray radiation conditions. These detectors also demonstrated significant improvements in X‐ray response parameters such as fast response times and low beam angle dependence owing to the unique structuring of the film morphology.

For typical applications as a curved detector, it is important to maintain high levels of bendability along with optimized detector performance. However, among the examples reported thus far for organic–inorganic hybrid direct conversion X‐ray detectors, there is very little understanding of how to tune both the microscopic and macroscopic properties of the film to achieve high curvatures (e.g., radius of a couple of millimeters as required in some medical imaging including keyhole operations). A key parameter that influences the physio‐mechanical, and electrical properties of a film is the molecular weight (MW) of the organic semiconductor used (P3HT). P3HT is generally known to show semi crystalline properties compared to its more modern analogues such as poly[N‐9“‐heptadecanyl‐2,7‐carbazole‐alt‐5,5‐(4”,7“‐di‐2‐thienyl‐2”,1“,3”‐benzothiadiazole)] ‐ PCDTBT which results in better charge transport over longer length scales.^[^ ^15^, ^16^ ^]^ Furthermore, for P3HT: PC_70_BM BHJ solar cells, P3HT MW has been correlated with charge carrier mobility, film morphology, and internal mechanical cohesion which has important implications on long term device reliability, functionality, and mass scale manufacturing of devices.^[^ ^17^, ^18^ ^]^ However, to the best of our knowledge there is no reported work for thick film (>10 *μ*m) detectors, in particular those used for X‐ray detection which demonstrates the impact of P3HT MW on i) the phase enrichment at the anode contact which is required to achieve ultra‐low dark currents,^[^ ^14^ ^]^ ii) phase separation for balanced extraction of X‐ray generated photo electrons and holes, and equally important; iii) its impact on the mechanical stability of curved (i.e., bendable) thick film hybrid detectors.

In this work, we demonstrate the important role of P3HT MW on the bendability characteristics of NP incorporated BHJ composite (NP‐BHJ) X‐ray detectors. We show that although all MW evaluated in this work (25 – 55 kDa) allows excellent detector response characteristics such as ultra‐low dark current, sensitivity, and response time etc., a higher P3HT MW is better suited for the curved X‐ray detector applications. Based on complementary characterization techniques including grazing incidence X‐ray scattering and nano‐mechanical studies, we show that a higher MW allows better relaxation of mechanical stresses. This is attributed to the formation of a higher number of intermolecular interactions and chain entanglements as well as the ability of longer polymer chains to form bridging ties between crystalline regions^[^ ^19^ ^]^ resulting in improved mechanical cohesion within the film. As a result, curved detectors fabricated with high molecular weights allow sensitivities of ≈ 0.17 *μ*C Gy^−1^ cm^−2^ to be maintained even when bent to a radius as small as 1.3 mm with less than 2.8% variation in X‐ray response after 100 bending cycles. This work emphasizes the influence of the organic semiconductor molecular weight on the nano‐mechanical properties of the NP‐BHJ film in designing curved detector technologies based on hybrid organic–inorganic semiconductor systems for direct conversion X‐ray detection.

## 2 Results and Discussion

**Figure 1:**
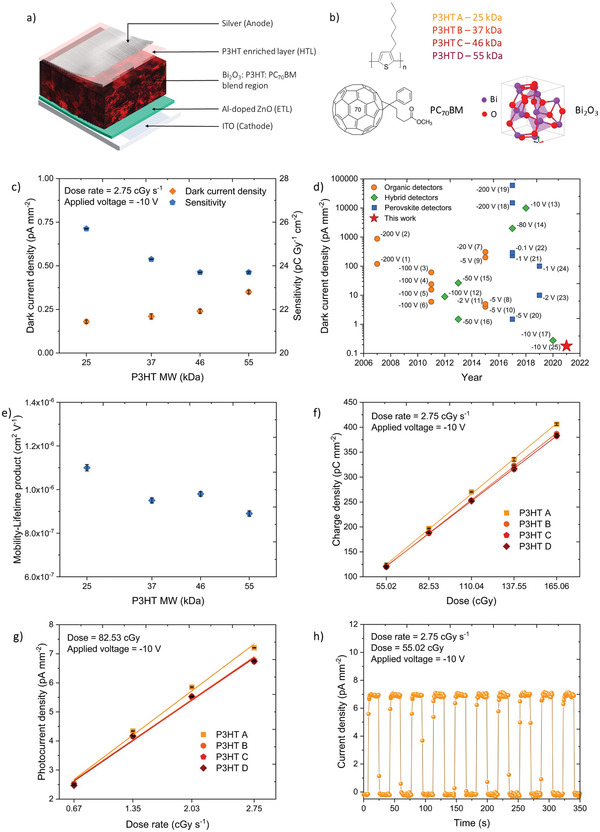
The effect of P3HT MW on NP‐BHJ detector performance. a) Schematic of the rigid detector architecture used in this work, b) Structure of X‐ray absorber Bi_2_O_3_, donor polymer P3HT, and PC_70_BM accepter, c) Dark current density and sensitivity of the NP‐BHJ detectors as a function of P3HT MW, d) Comparison of the dark current response of the organic detectors ((1)‐(2),^[^ ^24^ ^]^ (3)‐(6),^[^ ^25^ ^]^ (7),^[^ ^26^ ^]^ (8)‐(10),^[^ ^27^ ^]^ and (11)^[^ ^28^ ^]^), high‐Z NP sensitized hybrid detectors ((12),^[^ ^29^ ^]^ (13),^[^ ^12^ ^]^ (14),^[^ ^30^ ^]^ (15)‐(16)^[^ ^31^ ^]^), perovskite detectors ((18),^[^ ^32^ ^]^ (19),^[^ ^33^ ^]^ (20),^[^ ^34^ ^]^ (21)‐(22),^[^ ^35^ ^]^ (23),^[^ ^36^ ^]^ and (24)^[^ ^37^ ^]^), ultra‐low dark current detectors introduced in our previous work (17),^[^ ^14^ ^]^ and the P3HT A based detector fabricated in this work (25), e) *μτ* product of the NP‐BHJ detectors estimated using voltage dependence studies as a function of P3HT MW. f) Dose linearity and g) Dose rate linearity of the NP‐BHJ detectors with varying polymer MW, h) Reproducibility of the photocurrent response of the P3HT A based NP‐BHJ detectors under 10 repeated X‐ray exposures. Data points in Figure c), e), f), and g) are averaged over three separate detector measurements.

In order to understand the impact of P3HT MW on detector performance, rigid NP‐BHJ X‐ray detectors (**Figure** **1**a) were fabricated based on the hole transport layer free architecture as previously reported by us.^[^ ^14^ ^]^ The device stack used consists of glass/ indium tin oxide (ITO)/ zinc oxide (ZnO)/NP‐BHJ composite/silver (Ag). Four different molecular weights of P3HT (25, 37, 46, and 55 kDa labelled as P3HT A, B, C, and D, respectively) were selected for this study to cover a wide range of MW typically used for P3HT based optoelectronic devices (Figure 1b). Further details of the P3HT variants used are given in Table S1, Supporting Information. All the NP‐BHJ films were processed in a similar manner and maintained at a constant thickness of ≈ 55 *μ*m (further details are given under experimental methods). Initially, we investigated the influence of the P3HT MW on the dark current characteristics of the detectors. The detector dark current influences the lowest detectable dose, signal‐to‐noise ratio, and the dynamic range, all of which are significant figures‐of‐merit for both X‐ray dosimeters and imagers.^[^ ^20^ ^]^ As can be observed from Figure 1c, the dark current decreases with reducing polymer MW, where the detectors fabricated with P3HT A (MW = 25 kDa) display the lowest dark current of ≈ 0.18 pA mm^−2^ at −10 V which is 36% lower than that achieved in our previous reported work^[^ ^14^ ^]^ (Figure 1d). Furthermore, the rise in dark current with increasing applied bias was significantly lower for detectors fabricated with P3HT A, which only reached ≈ 1.6 pA mm^−2^ at −200 V, while the other detectors with P3HT B, C, D displayed dark currents in the range of 4 – 6 pA mm^−2^ (Figure S1, Supporting Information). However, regardless of the P3HT MW used, it is important to note that all detectors displayed low dark currents that is well within the industrial requirement of 10 pA mm^−2^ under an operational bias ranging from −10 to −200 V (where the latter is equivalent to a bulk electric field of ≈ 4 V µm^−1^) (Figure S1, Supporting Information). Following the characterization of dark diode properties, we evaluated the X‐ray photocurrent characteristics of the devices under a 70 kV X‐ray source. The sensitivity (*S*) of the detectors which is defined as the collected charge, per unit exposure of radiation, per unit area of incident radiation is operatively estimated from the slope of charge versus dose curve based on the equation^[^ ^21^ ^]^ below:

(1) $$S=\frac{Q}{\mathrm{DA}}=\frac{\underset{}{\int^{}}\left(I_{\mathrm{ON}}\left(t\right)-I_{\mathrm{OFF}}\right)\mathrm{dt}}{\mathrm{DA}}$$

where, *Q* represents the X‐ray generated charge, *I*_ON_ and *I*_OFF_ represent the current generated with and without X‐ray exposure, respectively, *D* is the X‐ray incident dose, and *A* is the sensitive area of the detector. Due to the thick nature of the detectors (≈ 55 µm thickness), larger MW is anticipated to be more preferable to maintain high hole mobilities,^[^ ^22^ ^]^ and thereby high detector sensitivities. However, contrary to this expectation we observed an enhancement in sensitivity with lowering of the P3HT MW, where the detectors based on P3HT A yielded a slightly higher sensitivity value of ≈ 26 nC Gy^−1^ cm^−2^ at −10 V (Figure 1c) which is an ≈ 8% improvement compared to detectors with higher MW where a sensitivity value of ≈ 24 nC Gy^−1^ cm^−2^ was observed at −10 V. We note that similar improvements in P3HT: PCBM solar cell device efficiencies have been reported for low P3HT MW,^[^ ^23^ ^]^ although the thickness of such devices is generally below 100 nm. A comparison of the sensitivity observed from the P3HT A based detectors with the sensitivities reported in the literature for stabilized amorphous selenium, cadmium zinc telluride, organic, hybrid, and perovskite detectors are shown in Figure S2, Supporting Information. Despite the relatively low thickness of these absorbers, NP‐BHJ detectors display a satisfactory performance compared to more established, state‐of‐the‐art detector technologies. In addition to sensitivity, the limit of detection (LoD) is another important detector parameter as lower LoD enables the use of reduced dosage for regular X‐ray examination, which ultimately eliminates the risk of exposure to high X‐ray doses (e.g., formation of secondary tumors as a result of high dose medical imaging). The LoD is defined as the equivalent dose rate required to produce a signal greater than three times the noise level (i.e., SNR >  3).^[^ ^20^ ^]^ We observed that the detectors based on P3HT A has a LoD of ≈ 1.5 *μ*Gy s^−1^ which is lower than the medical requirement for diagnostics (≈ 5.5 *μ*Gy s^−1^).^[^ ^20^ ^]^ This indicates the potential of NP‐BHJ detectors to be used for low dose imaging and dosimetry, thereby reducing potential risks for secondary tumors. A comparison of the LoD obtained for the P3HT A based detectors with the LoD reported in the literature for organic, perovskite, and hybrid detectors are shown in Figure S3, Supporting Information.

In an effort to understand the effect of MW on the charge transport characteristics of the NP‐BHJ composite, the mobility‐lifetime product (*μτ* product) was estimated by conducting voltage dependence studies on the detectors under a series of applied bias ranging from −10 to −200 V (Figure S4, Supporting Information). The relationship between charge extracted, applied bias, and the *μτ* product is given by the Hecht equation:^[^ ^38^ ^]^

(2) $$Q\;=Q_{0}\;\frac{\mu \tau V}{d^{2}}\left[1-exp\left(-\frac{d^{2}}{\mu \tau V}\right)\right]$$

where *Q* represents the total charge extracted, *Q*_0_ represents the asymptotic charge, *V* is the applied bias, *d* is the detector active layer thickness, *μ* is the carrier mobility, and *τ* is the carrier lifetime. By fitting the extracted charge versus applied bias curve to the Hecht equation, a significant improvement in the estimated *μτ* product was observed with reducing P3HT MW (Figure 1e), where the *μτ* product of the detectors fabricated with P3HT A displaying a value of ≈ 10^−6^ cm^2^ V^−1^ which is an order of magnitude higher than those obtained for higher MW detectors (≈ 10^−7^ cm^2^ V^−1^). Since the *μτ* product directly influences the charge collection efficiency of a detector, and subsequently the detector sensitivity, this behavior explains the significant improvement in the detector sensitivity when P3HT with lower MW is used. In addition to the above, regardless of the MW variation, NP‐BHJ detectors showed excellent dose linearity (linear relationship between charge and dose), dose rate linearity (linearity between photocurrent and dose rate) (Figure 1f,g). The detectors also displayed a highly reproducible X‐ray photocurrent response at low bias (−10 V) (Figure 1h) as well as at high bias conditions (−200 V) (Figure S5, Supporting Information), indicative of their stability under exposure to X‐rays and high bias conditions (electric fields).

**Figure 2:**
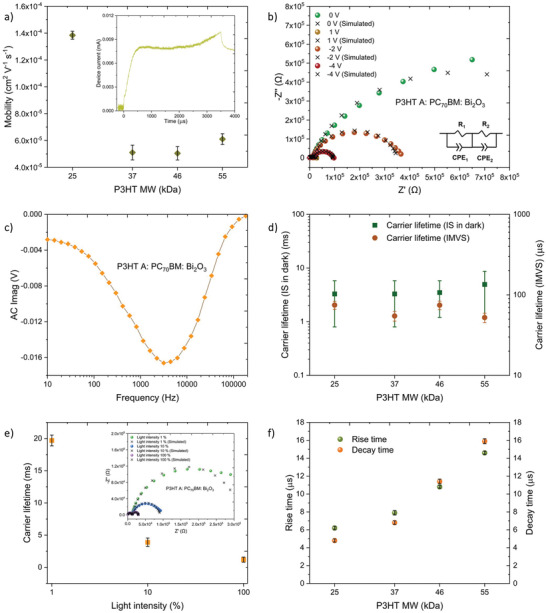
Influence of P3HT MW on charge transport characteristics of NP‐BHJ detectors. a) Variation of the carrier mobility estimated from photo‐CELIV method as a function of P3HT MW. Inset: Photoinduced charge transient of the NP‐BHJ detector fabricated with P3HT A, b) Nyquist plots of the NP‐BHJ detector fabricated with P3HT A under dark conditions when biased at +1, 0, −2, −4 V. The black color crosses ( × ) represent the fits for each bias calculated using the equivalent circuit shown in the inset. R_1_ and R_2_ are resistance components forming a parallel circuit with the constant phase elements CPE_1_ and CPE_2_, c) IMVS spectrum of the NP‐BHJ detector fabricated with P3HT A, d) Carrier lifetime estimated from IS and IMVS both as a function of P3HT MW, e) Carrier lifetime as a function of illumination light intensity of the NP‐BHJ detectors fabricated with P3HT A. Inset: Nyquist plots of the NP‐BHJ detector fabricated with P3HT A under illumination light intensity conditions of 1%, 10%, and 100% when biased at 0 V. The black color crosses ( × ) represent the fits for each light intensity condition calculated using the equivalent circuit model, f) Rise time and decay time estimated from TPC method both as a function of P3HT MW. Data points in Figure a), d), e) and f) are averaged over three separate detector measurements.

To better understand the influence of P3HT MW on the charge carrier mobility, we used the photo‐charge carrier extraction by linearly increasing voltage (photo‐CELIV) method. Inset of **Figure** **2**a displays a photoinduced charge transient of the detector fabricated with P3HT A recorded at a ramp rate of 1 V ms^−1^. Here, the charge mobility is estimated using the equation given below:^[^ ^39^ ^]^

(3) $$\mu =\frac{2d^{2}}{3Rt_{max}^{2}\left[1+0.36\frac{J_{max}-J_{o}}{J_{o}}\right]}$$

where *μ* represents the carrier mobility, *d* is the active layer thickness, *R* is the ramp rate, *J*_max_ represents the peak photocurrent, and the *J*_0_ represents the photocurrent plateau value at the end of the ramp. As shown in Figure 2a, a strong dependence of carrier mobility on the P3HT MW was observed with the highest mobility of ≈ 1.4 × 10^−4^ cm^2^ V^−1^ s^−1^ being achieved for devices based on P3HT A, which is an order of magnitude higher than the mobilities estimated for the higher MW detectors. Although it is not possible with CELIV analysis to discern whether the measured value is either hole or electron mobility, these results are in agreement with the mobility values reported for P3HT: PCBM solar cells^[^ ^23^ ^]^ where the carrier mobility improved with decreasing MW (from 48 to 24 kDa). As discussed later, this trend in higher mobilities at lower P3HT MW is due to the shorter chain length of the polymer which allows it to readily crystallize as we observe based on Grazing Incidence Wide Angle X‐ray Scattering (GIWAXS) studies.

To correlate the MW variation with the carrier lifetime of the NP‐BHJ detectors, we initially conducted impedance spectroscopy (IS) under dark conditions. The impedance spectra for each detector were acquired under four applied bias conditions (+1, 0, −2, −4 V). Irrespective of the different P3HT MW used, detectors displayed two semicircles on the Nyquist plot; one at the higher frequency regime and the other at lower frequency regime (Figure 2b and Figure S6, Supporting Information) which is characteristic of the NP‐BHJ system used.^[^ ^14^ ^]^ The impedance spectroscopy data were analyzed using the equivalent circuit model (inset of Figure 2b) which was successfully used previously^[^ ^14^ ^]^ to analyze the characteristics of these detectors. The carrier lifetime was estimated using the peak position of the larger semicircle in the low frequency regime where no noticable dependence of carrier lifetime on P3HT MW was noticed but spanned across the 3.3–4.9 ms range (Figure 2d). For each P3HT MW used, the *μτ* product estimated from the voltage dependence studies agrees well with the *μτ* product obtained independently from the photo‐CELIV (for carrier mobility) and IS (for carrier lifetime). The strong MW dependent behavior observed from the *μτ* product is explained by the similar trend portrayed for the carrier mobility, as carrier lifetime appeared to be insensitive to the MW variation. Furthermore, to understand the influence of increased carrier density on carrier lifetime, we conducted intensity modulated photovoltage spectroscopy (IMVS) measurements (Figure 2c and Figure S7, Supporting Information) under a white LED of 90% illumination intensity of the maximum (475 W m^−2^) and a modulation amplitude of 10%. The observed values are two orders of magnitude lower than the lifetimes estimated from IS method and ranged between 52 – 74 *μ*s for the detectors (Figure 2d). Such behavior is related to increased charge recombination rates under high carrier densities.^[^ ^40^ ^]^ This observation is further verified through intensity dependent IS under three different illumination light intensity conditions of 1%, 10%, and 100% (Inset of Figure 2e and Figure S8, Supporting Information) where the lifetime reduced with increasing light intensity (Figure 2e and Figure S9, Supporting Information).

To further relate the response characteristics of the detectors to the P3HT MW, time constants of the detectors were estimated using the transient photocurrent (TPC) method (Figure S10, Supporting Information). Each detector was illuminated with a 475 W m^−2^ white LED for a duration of 500 µs and the photocurrent responses were recorded at a bias of −10 V. The rise and decay times which were in the microsecond time scale appeared to be heavily influenced by the polymer MW where both time constants increased with the MW (Figure 2f). The detectors with the P3HT A showed the fastest rise and decay times of ≈ 6 and ≈ 4 *μ*s, respectively. Such fast response times are traced back to the higher charge carrier mobilities observed under lower MW, thereby leading to better charge extraction. It is also noteworthy to highlight that the decay time becomes shorter than the rise time for the lower MW detectors, suggesting that any defects tend to be energetically shallow when lower P3HT MW is used.^[^ ^20^, ^32^ ^]^

**Figure 3:**
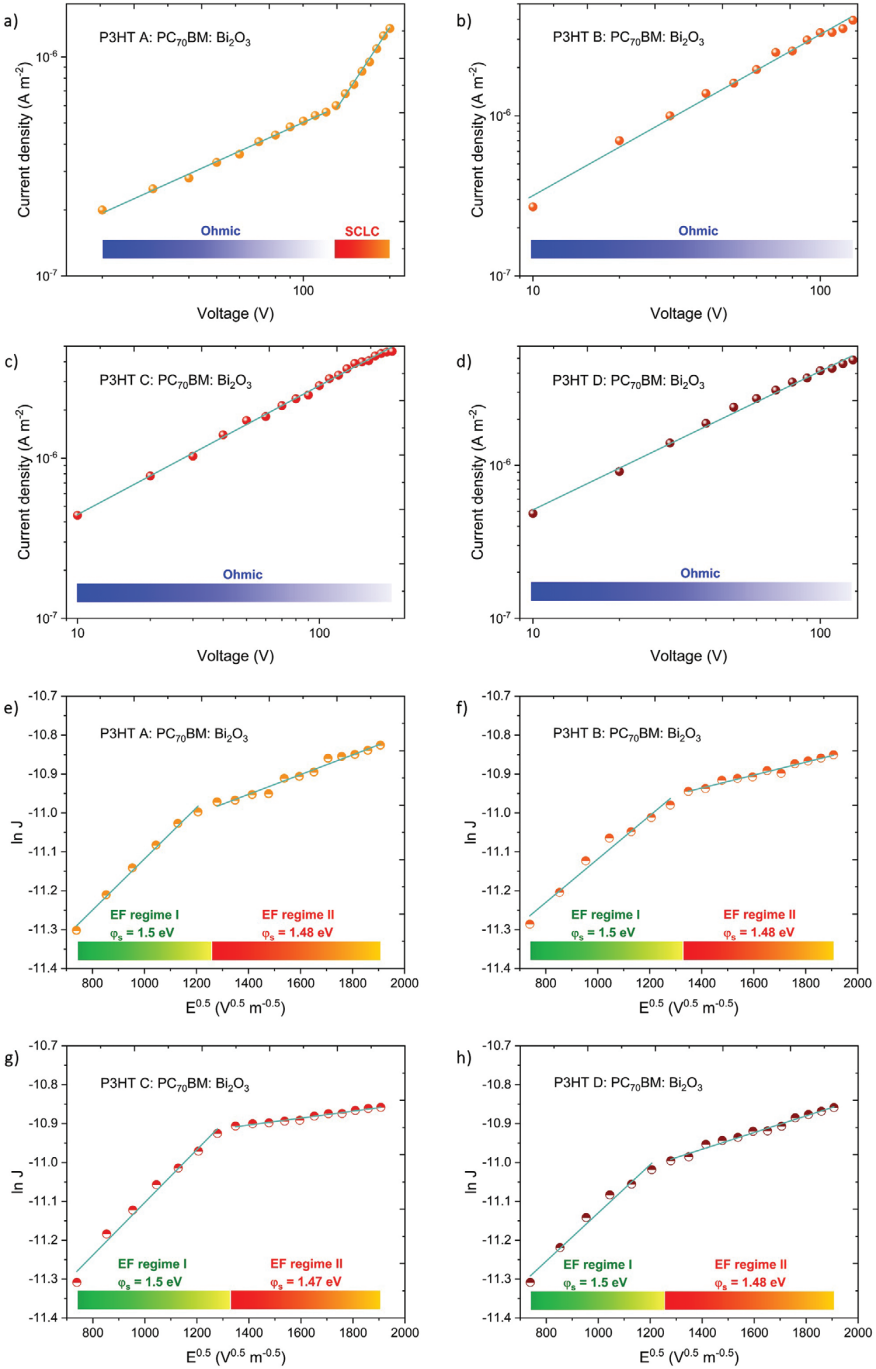
Charge conduction mechanisms prevalent within the NP‐BHJ detectors. Current density− Voltage plots under dark diode reverse bias conditions for NP‐BHJ detectors fabricated with a) P3HT A, b) P3HT B, c) P3HT C, and d) P3HT D. Linear and quadratic fittings are applied according to the SCLC model. ln (*J*) − *E* ^0.5^ plots under the X‐ray irradiation reverse bias conditions for NP‐BHJ detectors based on e) P3HT A, f) P3HT B, g) P3HT C, and h) P3HT D. The Schottky barrier type conduction is prevalent across two distinctive electric field (EF) regimes indicating two *φ*_*s*_ under X‐ray irradiation for the NP‐BHJ system.

To understand the charge conduction mechanisms prevalent within the NP‐BHJ detectors fabricated with different P3HT MW, we analyzed the dark diode and X‐ray photocurrent response characteristics of each detector under reverse bias conditions. Generally, charge conduction in such NP‐BHJ system can follow a number of mechanisms such as Fowler‐Nordheim tunnelling^[^ ^41^ ^]^ (Note S1 and Figures S11, S12, Supporting Information), space charge limited current (SCLC)^[^ ^42^ ^]^ (Note S2 and Figure S13, Supporting Information), Poole‐Frenkel^[^ ^41^ ^]^ (Note S3 and Figure S14 and S15, Supporting Information), and Schottky model^[^ ^43^ ^]^ (Note S4, Table S3, and Figure S16, Supporting Information). Based on the analysis carried out for the above mechanisms, the dark diode characteristics of the detectors based on P3HT A follow an Ohmic behavior at low electric fields (below 2.2 × 10^6^ V m^−1^) and trap free SCLC behavior at higher electric fields (**Figure** **3**a). However, the dark diode characteristics of the detectors based on P3HT B, C, and D display Ohmic behavior throughout the entire applied electric field (Figure 3b–d). Under X‐ray irradiation, response characteristics of all the NP‐BHJ detectors are dominated by Schottky barrier‐based conduction (A complete discussion on this analysis is given in Note S4, Supporting Information). Based on the fits to the Schottky barrier mechanism (Figure 3e–h), we observed two different Schottky barrier heights (*φ*_s_) with values of ≈ 1.5 eV within the electric field range of 0.55 × 10^6^ to 1.6 × 10^6^ V m^−1^ and ≈ 1.48 eV in the electric field range of 1.8 × 10^6^ to 3.6 × 10^6^ V m^−1^ (Table S2, Supporting Information). While the origins of the two barriers remain unclear at present, the observation of the influence of these barriers under X‐ray illumination is suggestive that this is likely to be due the formation of Schottky barriers between the X‐ray absorbing Bi_2_O_3_ NPs and the charge transporting medium P3HT and PC_70_BM (Figure S17, Supporting Information). However, a more careful, in‐depth study on the influence of these interfaces is required in order to identify the exact origins for these barriers.

**Figure 4:**
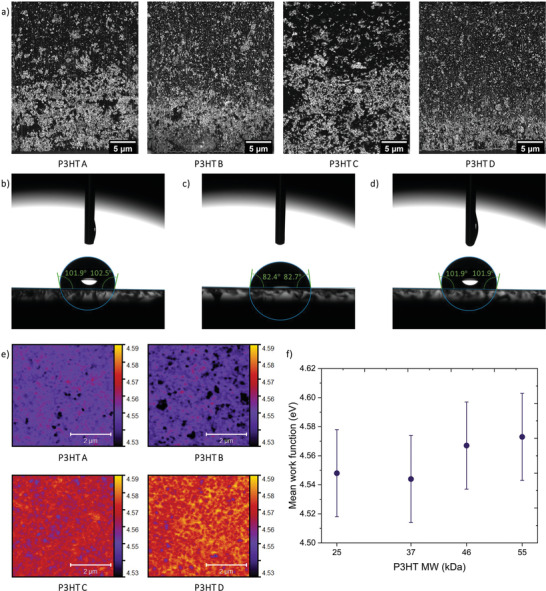
Impact of P3HT MW on NP‐BHJ film morphology. a) Backscattered electron cross‐sectional micrographs of the NP‐BHJ containing P3HT A, P3HT B, P3HT C, P3HT D illustrating the chemical compositional gradient. Surface free energy measurement of the b) P3HT A film, c) PC_70_BM film, d) P3HT A: PC_70_BM: Bi_2_O_3_ blend film using the sessile drop contact angle method, e) SKPM micrographs of the NP‐BHJ containing P3HT A, P3HT B, P3HT C, P3HT D depicting the work function distribution, f) work function variation of the NP‐BHJ film with P3HT MW. Data points in Figure f) are averaged across three separate 5 µm × 5 µm maps for different regions on the film.

Although MW impacts the carrier mobility of the NP‐BHJ detectors, variations in the response characteristics of the hybrid detectors could not be assigned solely to the changes in mobility, the active layer morphology is also likely to play a role. In order to identify the influence of the P3HT MW on the Bi_2_O_3_ NP distribution within the bulk of the film, cross sectional imaging of each NP‐BHJ film was carried out using dual‐beam microscopy (FIB‐SEM) (**Figure** **4**a). Similar to our previous work,^[^ ^14^ ^]^ we observed the enrichment of Bi_2_O_3_ NPs at the bottom of each film (bright regions in the images) leaving behind a more organic semiconductor rich capping layer (darker regions in the images). Furthermore, our previous work^[^ ^14^ ^]^ revealed that the vertical phase segregation induced enrichment of the P3HT polymer near the anode contact results in an inbuilt hole selective layer, leading to ultra‐low dark currents. As can be seen from the low dark current response characteristics of the NP‐BHJ detectors exploited in this study, it is apparent that a similar P3HT rich layer is formed near the anode contact, regardless of the MW variation. To verify this, we carried out surface free energy (*γ*) measurements (Figure 4b–d and Figure S18, Supporting Information) which allows the variation of the interaction parameter, *χ*, as described in the Flory‐Huggins theory^[^ ^44^ ^]^ to be studied. Under the Flory‐Huggins theory, the *χ* value represents the compatibility between two material components, where a higher *χ* value indicates a greater degree of phase separation within the material system.^[^ ^44^ ^]^ Since *χ* is proportional to the difference in *γ* of the components used in the material system,^[^ ^44^ ^]^ we estimated the *γ* of the films fabricated with different P3HT MW and PC_70_BM. The films fabricated from neat P3HT A, B, C, and D had a *γ* of 21.1, 21.3, 23, and 23.4 mN m^−1^, respectively which are significantly lower compared to the *γ* of the PC_70_BM film (32.8 mN m^−1^). This considerable difference between the *γ* of the two components indicates that there is a higher tendency for phase separation within the NP‐BHJ films and also segregation of P3HT closer to the film surface (to minimize *γ* of the overall film). Since the *γ* of the neat P3HT films did not indicate significant fluctuation with the varying MW, it is also reasonable to say that the degree of phase separation is similar for each NP‐BHJ film fabricated with different P3HT MW. In order to verify the formation of a P3HT enriched hole selective layer at the top film surface, *γ* of the NP‐BHJ films based on different P3HT MW were estimated. Favorably, each NP‐BHJ film based on P3HT A, B, C, and D displayed *γ* values of 21, 21.5, 22, and 22.9 mN m^−1^, respectively which is similar to the *γ* of the neat P3HT films establishing that regardless of the MW variation, P3HT forms an enriched phase near the top film surface. This is in agreement with the requirement to minimize the overall *γ* of the NP‐BHJ films.

Following the above, we carried out scanning Kevin probe microscopy (SKPM) to study the impact of P3HT MW on the surface potential of the NP‐BHJ film (Figure 4e), which provides a more detailed understanding of the nature of the electronic contact between the NP‐BHJ film and the Ag contact. As can be seen from Figure 4f, an average surface potential value of ≈ 4.6 eV was achieved from each NP‐BHJ film, indicating the possibility of forming an Ohmic contact with the Ag anode (work function 4.7 eV^[^ ^45^ ^]^). These observations are in agreement with the P3HT enrichment identified using the surface free energy measurement and also explains the ultra‐low dark currents observed in this architecture.

**Figure 5:**
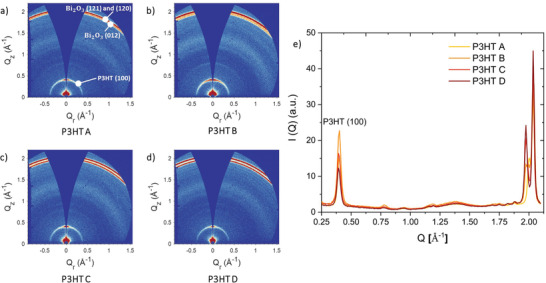
Effect of P3HT MW on the crystallinity of NP‐BHJ films. 2D GIWAXS detector images for the NP‐BHJ films fabricated with a) P3HT A, b) P3HT B, c) P3HT C, d) P3HT D, e) 1D azimuthally integrated line profiles of the NP‐BHJ film with various P3HT MW indicating the MW dependence of the intensity of the P3HT (100) peak.

For X‐ray detector applications, a higher crystallinity is preferred in order to achieve satisfactory charge extraction. On the other hand, for curved detector applications a higher crystallinity can result in mechanical failure as is often observed for inorganic crystalline semiconductors.^[^ ^5^ ^]^ In order to identify the most suitable P3HT MW in terms of the most favorable molecular packing for curved hybrid X‐ray detectors, GIWAXS measurements were carried out. The 2D GIWAXS detector images for each NP‐BHJ film are shown in **Figure** **5**a–d. Each film exhibited a peak at *Q_z_* ≈ 0.4Å^−1^ attributed to the P3HT (100) lamellar stacking^[^ ^46^ ^]^ indicating the crystallization of the P3HT phase. However, the NP‐BHJ film with the highest P3HT MW displayed the lowest (100) peak intensity, which appeared to increase when lower molecular weights are reached (Figure 5e). In previous studies,^[^ ^47^, ^48^ ^]^ the lower MW P3HT was reported to form separate crystalline regions whereas higher MW P3HT results in crystalline regions that are interconnected through bridging polymer chain molecules. In order to verify whether a similar phenomenon is occurring within the NP‐BHJ system, the average crystallite grain size was calculated using the Scherrer formula^[^ ^46^ ^]^ (Equation (4)) on the P3HT (100) peak in the 1D azimuthally integrated line profiles (Figure 5e):

(4) $$D\;=\frac{K\lambda }{\beta \cos\left(\theta \right)}\;$$

where *D* is the crystallite grain size, *K* is the dimensionless shape factor (0.94), *λ* is the X‐ray wavelength (0.134 nm), *β* is the full width at half maximum of the P3HT (100) peak in radians, and *θ* is the Bragg angle in radians. Regardless of the MW, each NP‐BHJ film displayed a similar P3HT crystallite size in the range of 16 – 18 nm. Since the crystallinity is lower at higher MW, this suggests that NP‐BHJ films fabricated with higher P3HT MW are more likely to have a larger number of amorphous regions which can form bridging between crystalline regions. These amorphous regions also increase intermolecular interactions and also raises the likelihood of interchain entanglements, which ultimately allows for a greater degree of plastic deformation before cohesive failure as the MW is increased.^[^ ^49^ ^]^ Overall, this indicates that the higher P3HT MW to be more suited for the fabrication of curved X‐ray detectors, since films fabricated with lower P3HT MW are more likely to display extremely brittle behavior upon handling.

**Figure 6:**
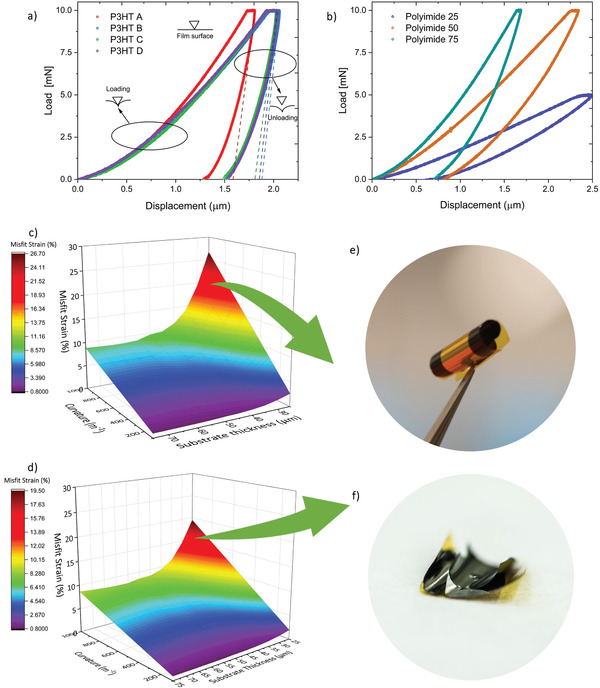
Role of P3HT MW and polyimide substrate thickness on the functionality of curved X‐ray detectors. Loading and unloading *P*–*h* curves for the a) NP‐BHJ films containing different P3HT MW and b) polyimide substrates of different thicknesses. Misfit strain as a function of both radius of curvature and substrate thickness for NP‐BHJ film based on c) P3HT A and d) P3HT D. Misfit strain between the film and substrate increases when thinner substrates and lower P3HT MW are used. Photograph indicating the tendency of NP‐BHJ films fabricated on the thinnest polyimide substrate (25 *μ*m thick) to e) curl excessively and, f) delaminate easily upon handling.

To develop curved X‐ray detectors, an understanding of the impact of P3HT MW and flexible substrate thickness on the nano‐mechanical properties of the NP‐BHJ films fabricated on flexible substrates is required. We selected polyimide as the flexible substrate due to its excellent radiation stability as evident from its use as a charge blocking layer in amorphous selenium X‐ray detectors.^[^ ^50^, ^51^ ^]^ The thickness of the substrate plays a key role on the ability to bend the NP‐BHJ X‐ray detectors without mechanical failure. In order to elucidate this, polyimide substrates with thicknesses lower (25 *μ*m), equal (50 *μ*m), and higher (75 *μ*m) than the NP‐BHJ layer were selected. To verify the effect of P3HT MW as well as substrate thickness on the nano‐mechanical properties (Young's modulus and hardness) of the curved X‐ray detectors consisting of NP‐BHJ films deposited on polyimide substrates, we utilized the nano‐indentation technique. Nanoindentation is a commonly used technique to characterize mechanical properties of various polymeric systems,^[^ ^52^, ^53^ ^]^ which involves the controlled deformation at the material surface whilst applying a given load and measuring the displacement over time. The mechanical properties at the film surface are estimated by analyzing the measured load–displacement curves (*P*–*h* curves). **Figure** **6**a,b illustrates the loading and unloading *P*–*h* curves for the NP‐BHJ films containing different P3HT MW and the polyimide substrates of different thicknesses, respectively. As observed in Figure 6a, the slope for both the loading and unloading curves (shown as dashed lines here) vary with the P3HT MW indicating the differences in the ordering of the NP‐BHJ blend morphology and thus the nano‐mechanical properties. A similar behavior was also observed from the polyimide substrates, suggesting that their nano‐mechanical properties are correlated to the substrate thickness (Figure 6b). The Young's modulus (*E*) of the NP‐BHJ films and the polyimide substrates were estimated based on the model proposed by Oliver and Pharr:^[^ ^54^ ^]^

(5) $$E\;=\;\frac{\left(1-\upsilon^{2}\right)}{\frac{1}{E_{r}}-\frac{\left(1-\upsilon_{i}^{2}\right)}{E_{i}}}$$

where *E_r_* is the reduced modulus given by $S\sqrt{pi}/2\sqrt{\overset{`}{A}}$, and *S* represents the contact stiffness (*dP*/*dh*) estimated from the slope of the initial unloading curve, and $\overset{`}{A}$ represents the real projected contact area of the indenter which is defined by the Berkovich tip and the contact depth (*h_c_*). υ and $\upsilon_{i}$ are the Poisson's ratio of the sample and the indenter (diamond). For polymeric films, the Poisson's ratio is taken as 0.3^[^ ^55^ ^]^ and the same value was used for both NP‐BHJ films and polyimide substrates during this analysis. The Poisson's ratio for the indenter is equal to 0.07.^[^ ^56^ ^]^ The *E_i_* in Equation (5) which represents the Young's modulus of the indenter was reported as 1141 GPa.^[^ ^56^ ^]^ The Young's modulus of each NP‐BHJ film fabricated from P3HT A, B, C, D was estimated to be 7.9, 6.7, 6.2, and 5.3 GPa, respectively. The lower Young's modulus observed at the higher MW indicates a reduction in stiffness for the NP‐BHJ films. In addition to the Young's modulus, we estimated the hardness (*H*) value of each film using:

(6) $$H=\frac{P_{\max}}{\hat{A}}$$

where *P*_max_ is the maximum value of applied load (10 mN) and $\hat{A}$ is the real projected contact area of the indenter which is given as 24.56$h_{c}^{2}$.^[^ ^55^ ^]^ A similar trend was observed for hardness where films fabricated with P3HT A, B, C, and D displayed hardness values of 138, 105, 110, and 111 MPa, respectively. Both the reduction in stiffness as well as hardness with increasing MW is in agreement with the trend of lower crystallinity observed for higher MW and also to the possibility of interchain P3HT entanglements as discussed previously based on the Scherrer analysis of the GIWAXS measurements. In addition to the above, the Young's modulus of polyimide substrates of different thicknesses (25, 50, and 75 *μ*m) were estimated to be 0.5, 1.2, and 2.5 GPa, respectively. Upon estimation of the Young's modulus for the NP‐BHJ films and the polyimide substrates, we proceeded to estimate the misfit strain (Δ) for the different combinations of NP‐BHJ films with different P3HT MW and the polyimide thicknesses at different radius of curvatures. For this purpose, we used the Timoshenko equation given below:^[^ ^57^ ^]^

(7) $$\Delta =\frac{kh\left(3{\left(1+m\right)}^{2}+\left(1+mn\right)\left(m^{2}+1/mn\right)\right)}{6{\left(1+m\right)}^{2}}$$

where *k* is the inverse of the radius of curvature, *h* is the total thickness of the sample,*m* = *t*_1_/*t*_2_ in which *t*_1_ and *t*_2_ are the thickness of the top and bottom layer, respectively.*n* = *E*_1_/*E*_2_ where *E*_1_ and *E*_2_ are the Young's modulus of the two layers. The misfit strain as a function of both curvature radius and substrate thickness for the NP‐BHJ films based on P3HT A, and P3HT D (i.e., the lowest and highest P3HT MW, respectively) are shown in Figure 6c,d, respectively. Misfit strain plots for the NP‐BHJ films based on remaining two P3HT MW are shown in Figure S19, Supporting Information. It should be noted that the misfit strain has been calculated only between the NP‐BHJ film and the polyimide substrate. Although for a full detector stack, an aluminum cathode and a ZnO electron transport layer are inserted between the NP‐BHJ film and the polyimide substrate, their influence on the misfit strain can be neglected due to the thin nature of these layers compared to the polyimide substrate and the NP‐BHJ film (both of which are ≈ 2 – 3 orders of magnitude thicker). Overall, we noticed that the misfit strain between the NP‐BHJ film and the polyimide substrate becomes considerably higher when lower substrate thicknesses and lower P3HT MW are used. Furthermore, we also carried out Grazing Incidence X‐ray Diffraction (GIXRD) measurements on NP‐BHJ films based on polyimide substrates, where we observe an improvement in P3HT (100) peak intensity compared to the rigid NP‐BHJ films based on glass substrates (Figure S20, Supporting Information). This is indicative that during the strain relief process (i.e., the curving of the NP‐BHJ/polyimide substrate) the P3HT undergoes further crystallization, indicating that amorphous semiconductors would be more desirable in terms of thick, curved detector applications. However, due to the natural bending radius displayed by curved films, the GIXRD needs to be interpreted with caution due to potential artefacts that can arise during the measurement process, and therefore requires more carefully detailed studies which are beyond the scope of this work.

**Figure 7:**
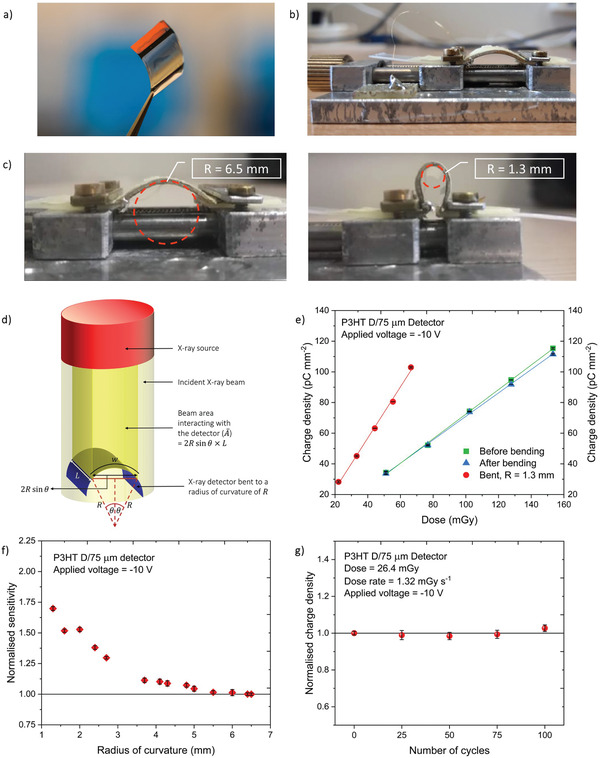
Bendability characteristics of NP‐BHJ detectors influenced by P3HT MW. a) Photograph of a curved NP‐BHJ X‐ray detector held with a tweezer, b) Experimental set‐up used for the bendability characterization of the NP‐BHJ detectors, c) Photograph of a curved NP‐BHJ X‐ray detector bent to a radius of curvature of 6.5 and 1.3 mm. d) Schematic diagram illustrating the variation of the beam area interacting with the detector with the bending radius of curvature, e) Charge density as a function of the incident dose for the P3HT D/75 μm detector measured before bending (green solid squares), during bending with a bending radius of 1.3 mm (red solid circles), and after bending (blue solid triangles) indicating dose linearity under each condition. f) Normalized sensitivity of the P3HT D/75 *μ*m detector as a function of bending radius indicating the threshold curvature radius limit for bendability, g) Normalized charge density of the P3HT D/75 *μ*m detector measured before bending, and after 25, 50, 75, and 100 bending cycles. Data points in Figure e), f), and g) are averaged over three separate detector measurements.

Based on these results, we expected the NP‐BHJ films fabricated on thinner flexible substrates to curl excessively and to delaminate easily upon handling. Therefore, from a design point of view, the use of higher P3HT MW and thicker flexible substrates was identified to be more suitable for the fabrication of curved hybrid detectors. To verify this, curved detectors were fabricated on polyimide substrates of three different thicknesses (25, 50, and 75 *μ*m) with a device layer stacking of polyimide/ aluminum (Al)/ zinc oxide (ZnO)/NP‐BHJ composite/silver (Ag). It should be noted that these detectors also adopt the hole transport layer free architecture previously reported by us.^[^ ^14^ ^]^ As predicted from the crystallinity and nano‐mechanical analysis, the mechanical behavior of the detectors was observed to be dependent on both the P3HT MW and the substrate thickness, where lower MW detectors tended to be extremely brittle during handling as well as detectors fabricated on thinner polyimide substrates appeared to curl excessively (Figure 6e) and delaminate easily from the substrates (Figure 6f). Due to this reason, only the NP‐BHJ films with P3HT C and D (MW = 55 and 46 kDa) fabricated on the 75 *μ*m polyimide substrate (Hereafter, P3HT C/75 *μ*m and P3HT D/75 *μ*m detector, respectively) and the NP‐BHJ film with P3HT D fabricated on polyimide 50 *μ*m substrate (Hereafter, P3HT D/50 *μ*m detector) were proven to be practicable for testing under X‐ray irradiation (**Figure** **7**a).

To assess the X‐ray detector response under different bending radii, the measurement setup shown in Figure 7b was used in conjunction with an X‐ray source operating at 40 kV. The detector was fixed between two PET substrates which are then clamped to the measurement setup to assure that the active area of the detector is fully subjected to the bending radius. We note that the detectors already demonstrated a natural radius of 6.5 mm in its pristine condition (Figure 7c) due to the inherent misfit strain between the NP‐BHJ layer and the polyimide substrate. The NP‐BHJ detectors irradiated under this initial condition displayed a box‐shaped, reproducible photocurrent response with a photo‐charge amplitude linearly increasing with the increasing incident dose. A sensitivity value of ≈ 0.08 *μ*C Gy^−1^ cm^−2^ was observed for P3HT D/75 *μ*m detector alongside a dark current response as low as ≈ 0.15 pA mm^−2^ when biased at −10 V. The detectors displayed excellent resistance to mechanical failure, even when bent to a radius as small as 1.3 mm (Figure 7c). This implies the conformability of these detectors to most of the curved surfaces indicating their potential use in a wide range of applications such as medical diagnostics. It should be noted that when a planar detector is used, the incident beam is interacting with the entire pixel area of the detector. However, when the detector is bent to a certain radius of curvature (*R*), the beam area interacting with the detector is reduced. For pixel with a length *L* ( = 8 mm for this study), width *w* ( = 8 mm for this study), and the angle (*θ*) between the beam axis and the pixel width (Figure 7d), the resulting reduced beam area is ($\bar{A}$) is given by:

(8) $$\bar{A}=2R\sin\theta \times L$$

where, *θ* is given by w/2R. This results in a lower dose being incident upon the detector when smaller curvature radius is reached. Initially, the sensitivity of these detectors was compared before bending, while bent (to a radius of 1.3 mm), and after bending (i.e., relaxation to its initial state) as shown in Figure 7e and Figure S21, Supporting Information. The sensitivity of each detector appeared to increase during bending as can be seen for the P3HT D/75 *μ*m detector where a 112% improvement in the sensitivity was observed (compared to an initial sensitivity of ≈ 0.08 *μ*C Gy^−1^ cm^−2^). However, the detector performance appeared to almost recover to its initial sensitivity after bending where a sensitivity of ≈ 0.076 *μ*C Gy^−1^ cm^−2^ was achieved. Furthermore, the variation in the detector sensitivity under a series of bending radii (from 6.4 to 1.3 mm) was compared to the sensitivity in pristine condition (Figure 7f). The sensitivity at each radius of curvature was normalized to that of the pristine condition. We noticed that up to a threshold radius of 3.7 mm, the detector does not display any significant deviation of sensitivity, whereas beyond that threshold limit the sensitivity improved considerably. For efficient X‐ray induced photocurrent generation, the NPs should ideally be surrounded by the organic semiconductor blend. This allows efficient transfer of holes and electrons generated within the single NP to the surrounding organic semiconductor charge transport pathways. As seen from the cross‐sectional micrographs, not only are the NPs segregated closer toward the substrate, but also these NPs show aggregation. During deformation, it is likely that the aggregated NPs are more separated from each other with the resulting volume being now occupied by the P3HT and PC_70_BM. This “temporary” rearrangement of the NP‐BHJ blend is likely to result in the enhancement in sensitivity as observed here. However, it should be noted that additional factors such as piezoelectricity in ZnO^[^ ^58^ ^]^ and the change in film thickness during the deformation process can result in localized electric field enhancements which can also lead to sensitivity enhancements. The mechanical robustness of the detectors was demonstrated by conducting dynamic bending up to 100 cycles down to a radius of 1.3 mm as shown in Figure 7g and Figure S22, Supporting Information. The charge density (which is proportional to the sensitivity) of each detector after 25, 50, 75, and 100 bending cycles was normalized to the charge density (or the sensitivity) before bending. The detectors did not display a significant degradation upon repeated bending, with a maximum deviation of around 2.7%, 2.4%, and 2.8% for charge density (or sensitivity) being observed for P3HT D/75 *μ*m, P3HT C/75 *μ*m, and P3HT D/50 *μ*m detectors, respectively.

## 3 Conclusion

In conclusion, we have demonstrated the importance of understanding the influence of the molecular weight of organic semiconductors in designing curved detectors based on the NP‐BHJ detector concept. The detectors fabricated with higher P3HT MW are shown to be more suitable for curved detector applications, demonstrating mechanical robustness up to bending radius as small as 1.3 mm, and a stable detector performance with less than 2.8% variation in performance under 100 repeated bending cycles. This impressive bendability characteristics at higher MW is attributed to the formation of a higher number of intermolecular interactions and chain entanglements and the tendency of longer polymer chains to form bridging between crystalline domains which ultimately increase the resistance to mechanical failure. Such detectors also demonstrated favorable detector response characteristics such as ultra‐low dark current response <1 pA mm^−2^ and sensitivity value of ≈ 0.17 *μ*C Gy^−1^ cm^−2^. This study indicates that by understanding and tuning fundamental properties such as polymer molecular weight, it is possible to realize curved hybrid detectors with the appropriate characteristics to function as radiation detectors in a wide range of applications.

## 4 Experimental Section

### Materials

Regioregular poly(3‐hexylthiophene‐2,5‐diyl) (P3HT) of 4002‐EE grade with four different molecular weights (25, 37, 46, 55 kDa) was purchased from Rieke Metals. [6,6]‐phenyl C71 butyric acid methyl ester (PC_70_BM) of purity >99% was purchased from Solenne. Bismuth oxide nanoparticles (Bi_2_O_3_) (with a *β* phase, tetragonal crystal structure; 38 nm diameter; surface area 18 m^2^ g^−1^) were purchased from Alfa Aesar. X‐ray detectors with different P3HT molecular weighs were fabricated by preparing the P3HT: PC_70_BM: Bi_2_O_3_ solution where 80 mg of each P3HT sample was mixed with 80 mg of PC_70_BM, 80 mg of Bi_2_O_3_ in 1 mL dichlorobenzene (DCB; 1 mL; anhydrous; Sigma‐Aldrich). The solution was stirred overnight followed by preheating at 60°C for 30 min before deposition of the films. The solution preparation was carried out in a N_2_ glove box (MBraun MB20G).

### Device Fabrication

Rigid devices were fabricated on ITO (In_2_O_3_: Sn) glass substrates (15 mm × 15 mm, 15 Ω per square, Luminescence Technology Corp.) and curved devices were fabricated on polyimide substrates (15 mm  ×  15 mm, RS Components) of three different thicknesses (25, 50, and 75 *μ*m), with 120 nm aluminum layer deposited as the cathode using thermal evaporation. An electron transporting aluminum‐doped zinc oxide (ZnO) NP dispersion (Sigma‐Aldrich) layer was spin coated in air (3000 rpm for 30 s) and annealed at 80°C for 10 min to give a thickness of 40 nm. P3HT: PC_70_BM: Bi_2_O_3_ solution was then drop casted to give a film thickness of 55 *μ*m. Devices were annealed (at 60°C) for ≈ 60 min in air, until a relatively dry layer was obtained. After the low temperature annealing process, devices were annealed at 140°C for 10 min in a N_2_ glove box (MBraun MB20G). Devices were kept under vacuum at a pressure of less than 3 × 10^−3^ mbar for 48 h to remove any residual solvent. This was followed by the deposition of the silver anode (≈ 120 nm) by thermal evaporation.

### X‐Ray Irradiation and Characterization

For each detector, three measurements were recorded under each irradiation condition. Detector response was characterized under soft X‐ray radiation from:

1. A 70 kV microfocus X‐ray source (Hamamatsu L6732‐01) under a dose rate range of 0.67 – 2.75 cGy s^−1^. A Keithley 2410 source measurement unit was used for recording the electrical characteristics.
2. A 40 kV microfocus X‐ray source (Hamamatsu L12161‐07) under a dose rate range of 1.0 – 5.4 mGy s^−1^. A Keithley 2600 source measurement unit was used for recording the electrical characteristics.

### Bendability Characterization

Curved X‐ray detectors were fabricated on polyimide substrates as mentioned earlier. Bendability test on the devices were conducted using an in‐house built uniaxial stretcher in combination with a Python program for position control (actuator speed 1 mm s^−1^). A Keithley 2600 source measurement unit was used for recording the electrical characteristics.

### Nanoindentation

Glass/ITO/NP‐BHJ films and polyimide films (15 mm  ×  15 mm) were prepared as stated earlier. The Young's modulus and Hardness of each film were measured by an Alemnis Standard Assembly nanoindenter (Alemnis AG). The nanoindentation tests were conducted with a Berkovich (three‐side pyramid) diamond indenter which applied constant loading and unloading rate of 0.2 mN s^−1^ after a load threshold of 0.5 mN. The maximum load was set to 10 mN to ensure a displacement lower than 10% of the film thickness, which was held for 10 s in order to check if the displacement under steady load was lower than ± 10 nm min^−1^.

### GIWAXS

Glass/ITO/NP‐BHJ samples were prepared as stated earlier. GIWAXS measurements were performed using a Xeuss 2.0 (Xenocs, France) system equipped with a liquid gallium MetalJet source (Excillum, Sweden) which provides a 9.243 kV X‐ray beam. The beam was collimated to a spot with a lateral dimension of 400 µm on the sample. A Pilatus3R 1M 2D detector (Dectris, Switzerland) placed at ≈307 mm from the sample was used to obtain the diffraction images with both the sample chamber and flight tubes held under vacuum to remove background air scatter. Calibration of the sample‐to‐detector distance was carried out using a silver behenate calibrant in transmission geometry. This data was corrected, reduced and reshaped using the GIXSGUI MATLAB toolbox. Parameters for the Scherrer formula were extracted from Gaussian curve fittings of the P3HT (100) peak using OriginPro software.

### GIXRD

Glass/ITO/NP‐BHJ and Polyimide/ NP‐BHJ samples were prepared as stated earlier. GIXRD crystallographic data for the samples was collected using a Panalytical X'Pert PRO diffractometer using a GI thin film bracket stage and monochromatic Cu *Kα*_1_ and Cu *Kα*_2_ radiation of wavelengths 1.54056 and 1.54439 Å (40 mA, 45 kV), sample offsets were callibrated to 0°, incident angle (omega) of 0.3, a step size of 0.02° was used.

### Photo‐CELIV, IS, IMVS, IDIS, and TPC

The Photo‐CELIV, impedance spectroscopy, intensity modulated photovoltage spectroscopy, intensity dependent impedance spectroscopy, and transient photocurrent measurements were conducted using the Paios 4 all‐in‐one test platform by FLUXiM (Paios 4, Platform for all‐in‐one characterization of solar cells and OLEDs, Fluxim AG 2019, https://www.fluxim.com/paios). Detectors were fabricated using the same method described for X‐ray response characterization. The pixel area was reduced to 3 mm^2^ in order to minimize capacitive effects that can influence device characteristics.

### Drop Shape Analysis

Glass/ITO/NP‐BHJ, Glass/ITO/P3HT, and Glass/ITO/PC_70_BM samples were prepared as stated earlier. The contact angle was determined from the shadow image of a sessile drop of water by using the drop shape analyzer (DSA25, KRÜSS GmbH). The surface free energy of each film was estimated from the ADVANCE software by using three contact angle measurements.

### Cross Sectional Imaging

Samples were prepared by fabrication of each NP‐BHJ film on the ITO coated glass substrates as described earlier. Cross sectional morphology of the NP‐BHJ film was examined using a FERA3; TESCAN dual beam/focused ion beam scanning electron microscope under an accelerating voltage of 5 kV.

### SKPM

Glass/ITO/NP‐BHJ samples were prepared as stated earlier. The surface potential of each NP‐BHJ film was evaluated by amplitude‐modulated 2‐pass scanning Kelvin probe microscopy (Combiscope 1000; AIST‐NT). The work function of the tip was calibrated on highly ordered pyrolytic graphite (HOPG) before and after the measurement on the NP‐BHJ film to account for drift due to adsorbed water. The measurements were conducted in dry nitrogen, with oxygen and water below 1 ppm.

## Conflict of Interest

K.D.G.I.J. and S.R.P.S. are inventors on a patent (Direct Conversion Radiation Detector, International Publication Number: WO2018/078372A1) which is assigned to SilverRay Ltd.

## Supporting information

Supporting Information

## Data Availability

The data that support the findings of this study are available from the corresponding author upon reasonable request.
