# Supplementary material for: Molecular Weight Tuning of Organic Semiconductors for Curved Organic–Inorganic Hybrid X‐Ray Detectors
Source: Adv Sci (Weinh). 2021 Nov 10;9(2):2101746. doi: 10.1002/advs.202101746 (PMC8805550; doi:10.1002/advs.202101746)
Supplement: Supplementary file 1 — Supporting Information [file ADVS-9-2101746-s001.pdf]

© 2021 Wiley-VCH GmbH

ADVANCED SCIENCE
Open Access

Supporting Information

for *Adv. Sci.,* DOI: 10.1002/advs.202101746

Molecular weight tuning of organic semiconductors for curved organic-inorganic hybrid X-ray detectors

*M. Prabodhi A. Nanayakkara, Mateus G. Masteghin, Laura Basiricò, Ilaria Fratelli, Andrea Ciavatti, Rachel C. Kilbride, Sandra Jenatsch, Thomas Webb, Filipe Richheimer, Sebastian Wood, Fernando A. Castro, Andrew J. Parnell, Beatrice Fraboni, K. D. G. Imalka Jayawardena, S. Ravi P. Silva**

© Copyright 2020. WILEY-VCH GmbH.

Supporting Information

Molecular weight tuning of organic semiconductors for curved organic-inorganic hybrid X-ray detectors

*M. Prabodhi A. Nanayakkara, Mateus G. Masteghin, Laura Basiricò, Ilaria Fratelli, Andrea Ciavatti, Rachel C. Kilbride, Sandra Jenatsch, Thomas Webb, Filipe Richheimer, Sebastian Wood, Fernando A. Castro, Andrew J. Parnell, Beatrice Fraboni, K. D. G. Imalka Jayawardena, S. Ravi P. Silva**

M. P. A. Nanayakkara, M. G. Masteghin, Thomas Webb, Dr K. D. G. I. Jayawardena, Prof. S. R. P. Silva
Advanced Technology Institute, Department of Electrical and Electronic Engineering, University of Surrey, Guildford, Surrey, GU2 7XH, United Kingdom.

Dr L. Basiricò, Dr I. Fratelli, Dr A. Ciavatti, Prof. B. Fraboni
Department of Physics and Astronomy, University of Bologna, Viale Berti Pichat 6/2, Bologna 40127, Italy.
National Institute for Nuclear Physics, INFN section of Bologna, Bologna, Italy.

R. C. Kilbride, Dr A. J. Parnell
Department of Physics and Astronomy, University of Sheffield, Hicks Building, Sheffield, S3 7RH, United Kingdom.

Dr S. Jenatsch
FLUXiM AG, Katharina-Sulzer-Platz 2, 8400 Winterthur, Switzerland.

F. Richheimer, Dr S. Wood, Dr F. A. Castro
National Physical Laboratory, Teddington, Middlesex, TW11 0LW, United Kingdom.

Note S1. Fowler-Nordheim tunnelling model.

This mechanism involves charge tunnelling through a potential barrier which generally reduces upon application of an electric field (in the range of 10^5 – 10^6 V m^{-1} or higher). Assuming that the potential barrier is triangular, and the electric field is uniform, the tunnelling current density (J) is given as[41]:

$$J \propto E^2 exp\left(-\frac{\kappa}{E}\right) \quad (1)$$

where E is the electric field and κ is given by $8\pi\sqrt{2m^*\varphi^3}/3eh$ and φ represents barrier height, m^* is the effective mass of the charge carrier, e is the electron charge, and h is the Planck's constant. In order to confirm whether the charge conduction is governed by the Fowler-Nordheim model, dark diode and X-ray photocurrent response characteristics were plotted as $ln(JE^{-2})$ vs E^{-1} (Figure S11 and S12). For the Fowler-Nordheim characteristics to be prevalent, the gradient of the $ln(JE^{-2})$ vs E^{-1} plots should be negative. Since all the detectors display a positive gradient, this indicates that Fowler-Nordheim is a less dominant charge conduction mechanism for these detectors in reverse bias.

Note S2. Space charge-limited current model

The SCLC model is existent when electric fields higher than 10^5 – 10^6 V m^{-1} are applied. Initially the concentration of the background carriers is higher than the injected carriers, hence the charge conduction follows an Ohmic behaviour where the gradient of the log $(J) -$ log (V) is equal to 1. This situation is explained as given below[42]:

$$I = en\mu \frac{V}{d} \tag{2}$$

where I is the current, V is the applied bias, e is the electron charge, n is the dominant carrier density, μ is the charge carrier mobility, and d is the active layer thickness. Upon increasing applied bias, concentration of injected carriers become higher than the background carriers. This results in the traps present within the system being filled. If the deep traps are present, the current density-voltage relationship follows the form of $J \propto V^n$ where $n > 2$. After reaching a specific applied bias, all the traps present within system become filled, therefore resulting in the trap free SCLC regime which is described as[42]:

$$J = \frac{9}{8}\mu\varepsilon_r\varepsilon_0\frac{V^2}{d^3} \tag{3}$$

where J is the current density, V is the applied bias, μ is the charge carrier mobility, ε_r is the relative permittivity and ε_0 is the permittivity in free space or vacuum, and d is the active layer thickness. During the trap free SCLC regime, the gradient of the $\log(J) - \log(V)$ is equal to 2.

Note S3. Poole-Frenkel model.

This mechanism is only applicable at electric fields higher than $10^5 – 10^6$ V m^{-1} and involves extraction of charge trapped in a potential well upon application of a sufficient electric field and is explained using the equation given below[41]:

$$J = Eexp\left(-\frac{(\phi - e\beta_{PF}E^{0.5})}{kT}\right) \quad (4)$$

where J is the current density, E is the electric field, ϕ is the potential energy of the traps, T is the absolute temperature, e is the electron charge, k is the Boltzmann constant, and β_{PF} is given as the $\sqrt{e/\pi\varepsilon_0\,\varepsilon_r}$. The ε_r represents relative permittivity and ε_0 is permittivity in free space or vacuum. In order to confirm whether extraction of such charge trapped in a potential well assists charge conduction in this NP-BHJ system, dark diode and X-ray photocurrent response characteristics were plotted as $ln(JE^{-1})$ vs $E^{0.5}$ (Figure S14 and S15). One of the main features which indicates that Poole-Frenkel mechanism is responsible for charge conduction within a material system is a linear behaviour between $ln(JE^{-1})$ vs $E^{0.5}$ with a positive gradient. However, under both dark diode and X-ray irradiation conditions, all the detectors indicate a negative gradient which indicate that Poole-Frenkel mechanism is less dominant under those situations.

Note S4. Schottky model

The Schottky model is explained using the equation given below[41]:

$$J = A^{*}T^{2}exp\left(\frac{-(\varphi_{S}-e\beta_{S}E^{0.5})}{kT}\right) \quad (5)$$

where J is the current density, E is the electric field, φ_s is the Schottky barrier height, A^* is the modified Richardson constant, T is the absolute temperature, e is the electron charge, k is the Boltzmann constant, and β_s is given as the $\sqrt{e/4\pi\varepsilon_0\,\varepsilon_r}$. Here, ε_r is relative permittivity and ε_0 is permittivity in free space or vacuum.

The relative permittivity of P3HT: $PC_{70}BM$ BHJ films are reported to be around 3.5,[59] whereas it ranges from 28 to 52 for β-Bi_2O_3.[60] Based on the models available[60] for estimating the ε_r of such blend films, it is apparent that the net ε_r should lie in between that of the BHJ system and the β-Bi_2O_3. In order to confirm whether the charge conduction is governed by the Schottky barrier model, dark diode and X-ray photocurrent response characteristics were plotted as $ln(J)$ vs $E^{0.5}$ (Figure 3e-h and Figure S16). Under X-ray irradiation conditions, the charge conduction is dominated by Schottky barrier model as the ε_r values estimated using the linear fits under X-ray irradiation conditions appeared to be in good agreement with the expected values for this NP-BHJ system.

Table S1. Summary of the properties of each P3HT MW as given by the manufacturer

P3HT Sample	MW [kDa]	M_n [kDa]	PDI
A	25	19.4	1.7
B	37	18.5	2
C	46	24.7	2.3
D	55	22	2.5

Table S2. ε_r and φ_s (eV) estimated using the best fit lines for the X-ray photocurrent response characteristics of the NP-BHJ X-ray detectors fabricated with different P3HT MW. The two different electric field (EF) regimes, where EF(I) is from 0.55×10^6 V m^{-1} to 1.6×10^6 V m^{-1} and EF(II) is from 1.8×10^6 V m^{-1} to 3.6×10^6 V m^{-1} is chosen based on the best linear fits.

Detector	$\boldsymbol{\varepsilon_r}$		$\boldsymbol{\varphi_s}$ (eV)	
	EF(I)	EF(II)	EF(I)	EF(II)
P3HT A: $PC_{70}BM$: Bi_2O_3	5.19	30.99	1.5	1.48
P3HT B: $PC_{70}BM$: Bi_2O_3	7.20	78.16	1.5	1.48
P3HT C: $PC_{70}BM$: Bi_2O_3	4.13	285.18	1.5	1.47
P3HT D: $PC_{70}BM$: Bi_2O_3	5.88	46.67	1.5	1.48

Table S3. ε_r and φ_s (eV) estimated using the best fit lines for the dark diode characteristics of the NP-BHJ X-ray detectors fabricated with different P3HT MW. The two different electric field regimes, where EF(I) is from 0.18×10^6 V m^{-1} to 2.2×10^6 V m^{-1} and EF(II) is from 2.4×10^6 V m^{-1} to 3.6×10^6 V m^{-1} is chosen based on the best linear fits.

Detector	$\boldsymbol{\varepsilon_r}$		$\boldsymbol{\varphi_s}$ (eV)	
	EF(I)	EF(II)	EF(I)	EF(II)
P3HT A: $PC_{70}BM$: Bi_2O_3	1.62	0.35	1.68	1.77
P3HT B: $PC_{70}BM$: Bi_2O_3	0.42	-	1.66	-
P3HT C: $PC_{70}BM$: Bi_2O_3	0.42	1.24	1.65	1.62
P3HT D: $PC_{70}BM$: Bi_2O_3	0.25	1.54	1.67	1.60

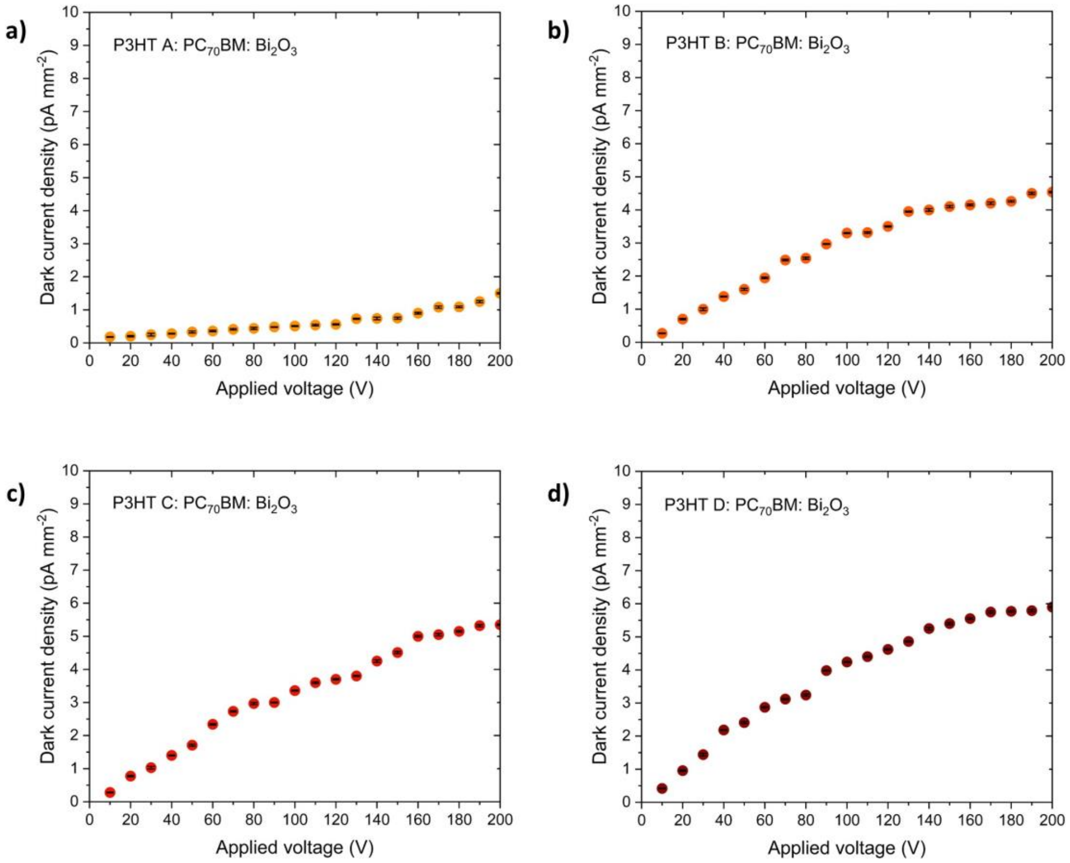

Figure S1. Dark current density as a function of applied bias of the NP-BHJ detectors fabricated with a) P3HT A, b) P3HT B, c) P3HT C, d) P3HT D.

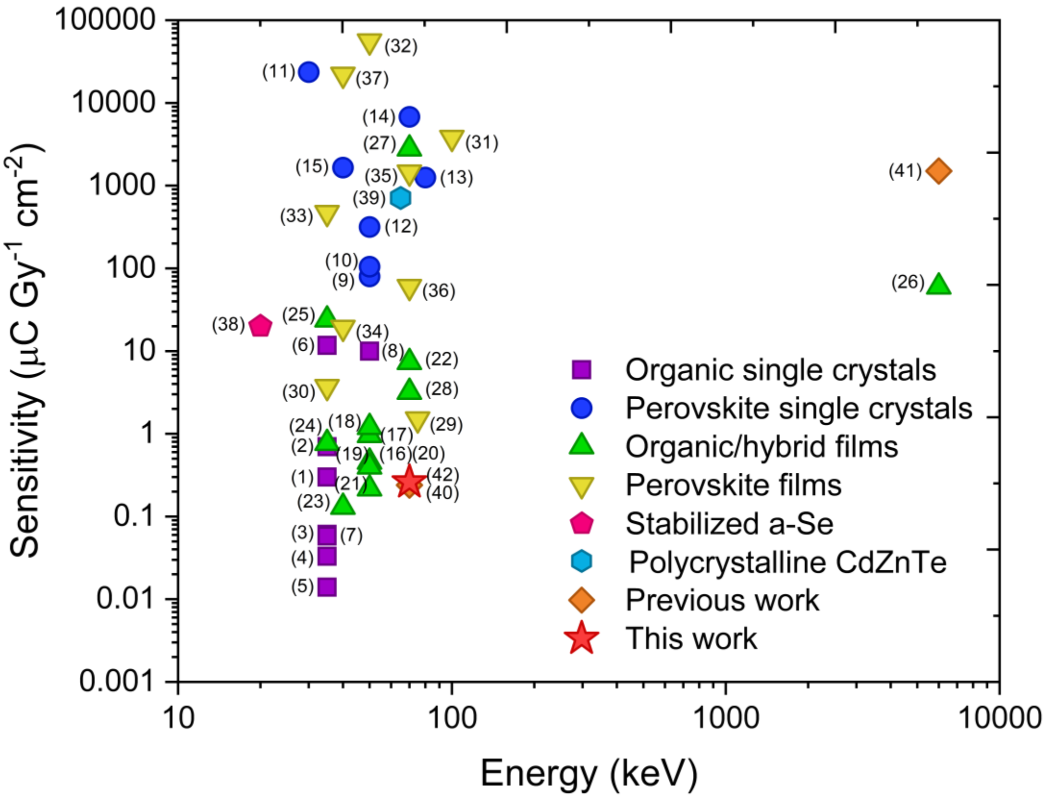

Figure S2. Comparison of the sensitivity of the organic single crystals ((1),[61] (2)-(3),[62] (4)-(5),[27] (6),[26] (7),[63] (8)[64]), perovskite single crystals ((9),[65] (10),[34] (11),[66] (12),[67] (13),[68] (14),[69] and (15)[70]), organic/hybrid films ((16)-(17),[24] (18),[71] (19),[25] (20),[29] (21),[31] (22),[21] (23),[72] (24),[73] (25),[30] (26),[74] (27),[38] and (28)[75]), perovskite films ((29),[76] (30),[77] (31),[32] (32),[78] (33),[36] (34),[79] (35),[80] (36),[81] and (37)[82]), stabilized amorphous Selenium (a-Se) detectors (38),[83] polycrystalline cadmium zinc telluride (CdZnTe) detectors (39),[84] ultra-low dark current detectors introduced in our previous work (40)-(41)[14], and the P3HT A based detector fabricated in this work (42).

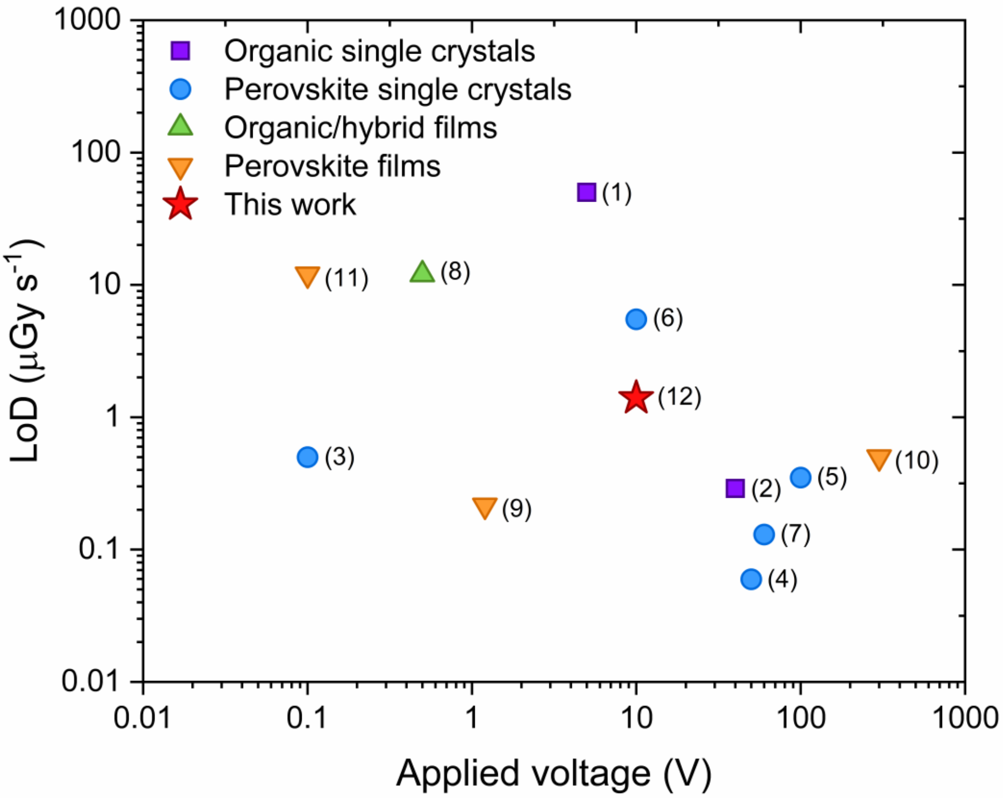

Figure S3. Comparison of the LoD of the organic single crystals ((1)[26] and (2)[64]), perovskite single crystals ((3),[65] (4),[34] (5),[66] (6),[69] and (7)[70]), organic/hybrid films ((8)[75]), perovskite films ((9),[78] (10),[80] and (11)[81]), and the P3HT A based detector fabricated in this work (12).

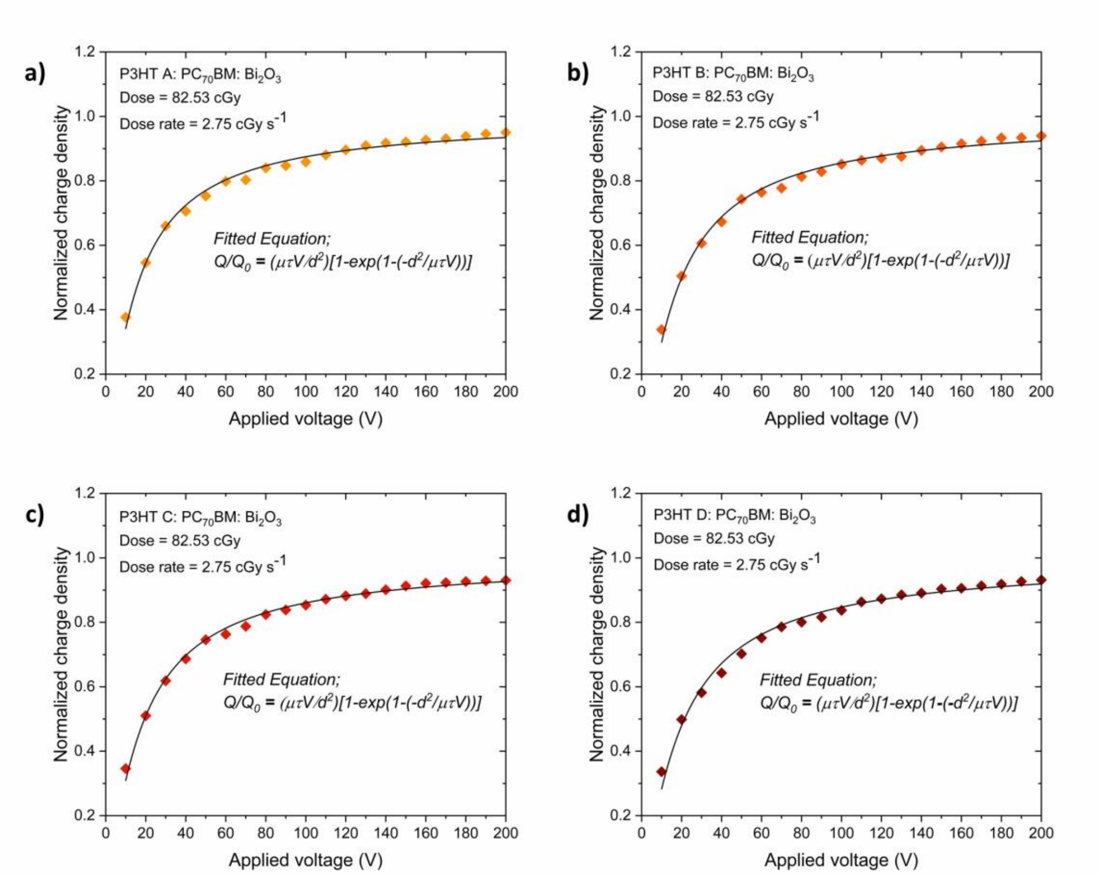

Figure S4. Voltage dependence together with the Hecht fit (R^2 > 0.9998) of the NP-BHJ detectors fabricated with a) P3HT A, b) P3HT B, c) P3HT C, d) P3HT D.

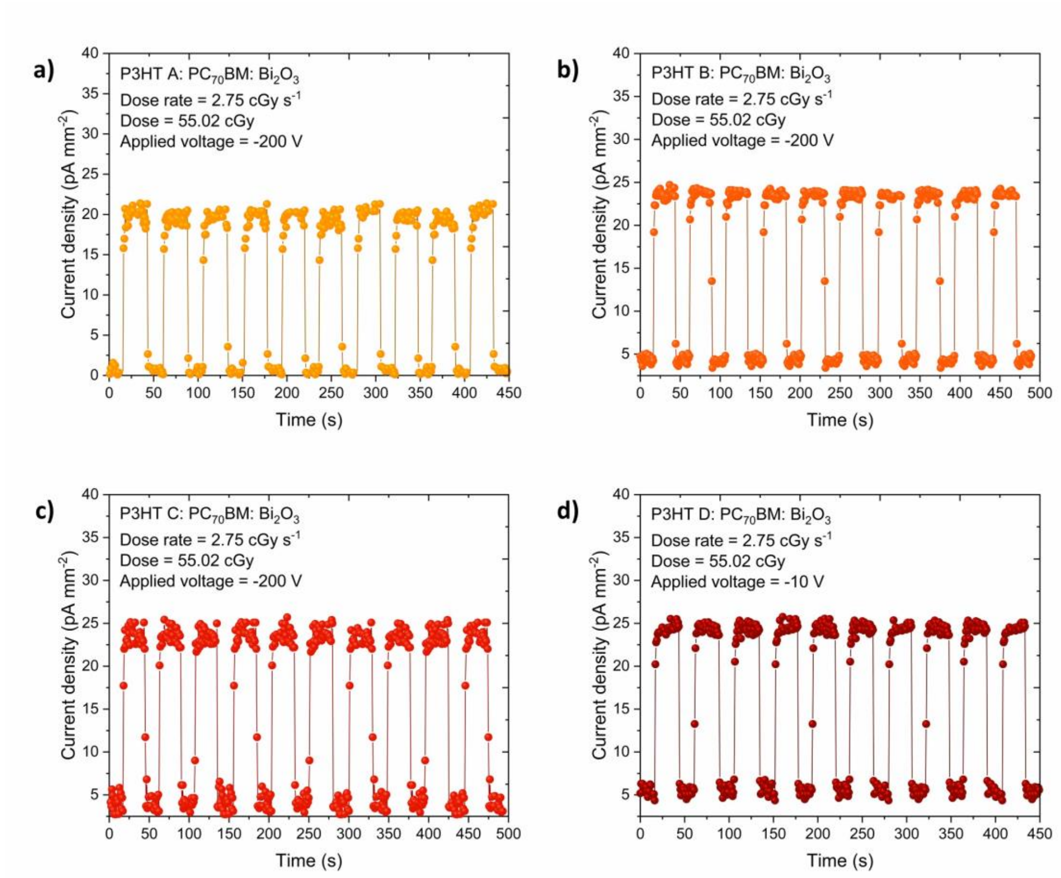

Figure S5. Reproducibility of the photocurrent response of the NP-BHJ detectors fabricated with a) P3HT A, b) P3HT B, c) P3HT C, d) P3HT D under an applied bias of -200 V.

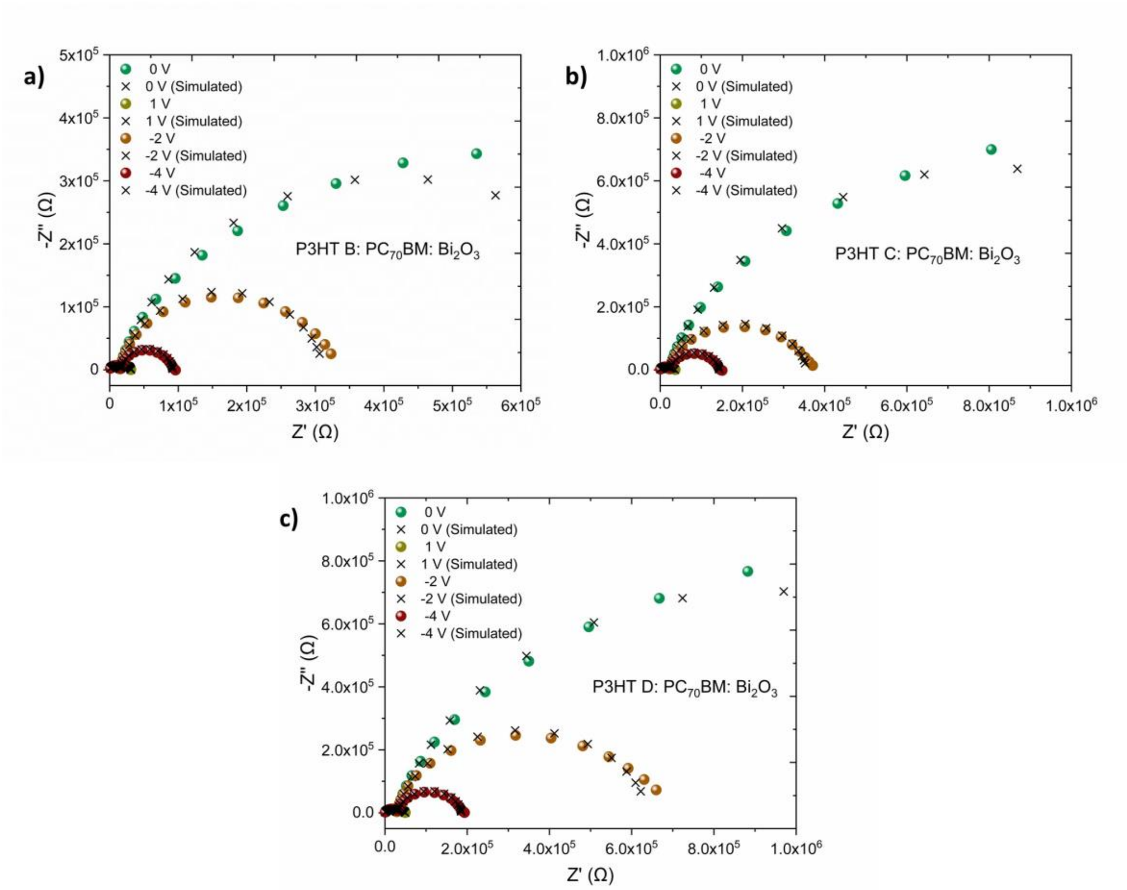

Figure S6. Nyquist plots of the NP-BHJ detector fabricated with a) P3HT B, b) P3HT C, c) P3HT D under dark conditions when biased at +1, 0, -2, -4 V. The black colour crosses (×) represent the fits for each bias calculated using the equivalent circuit model.

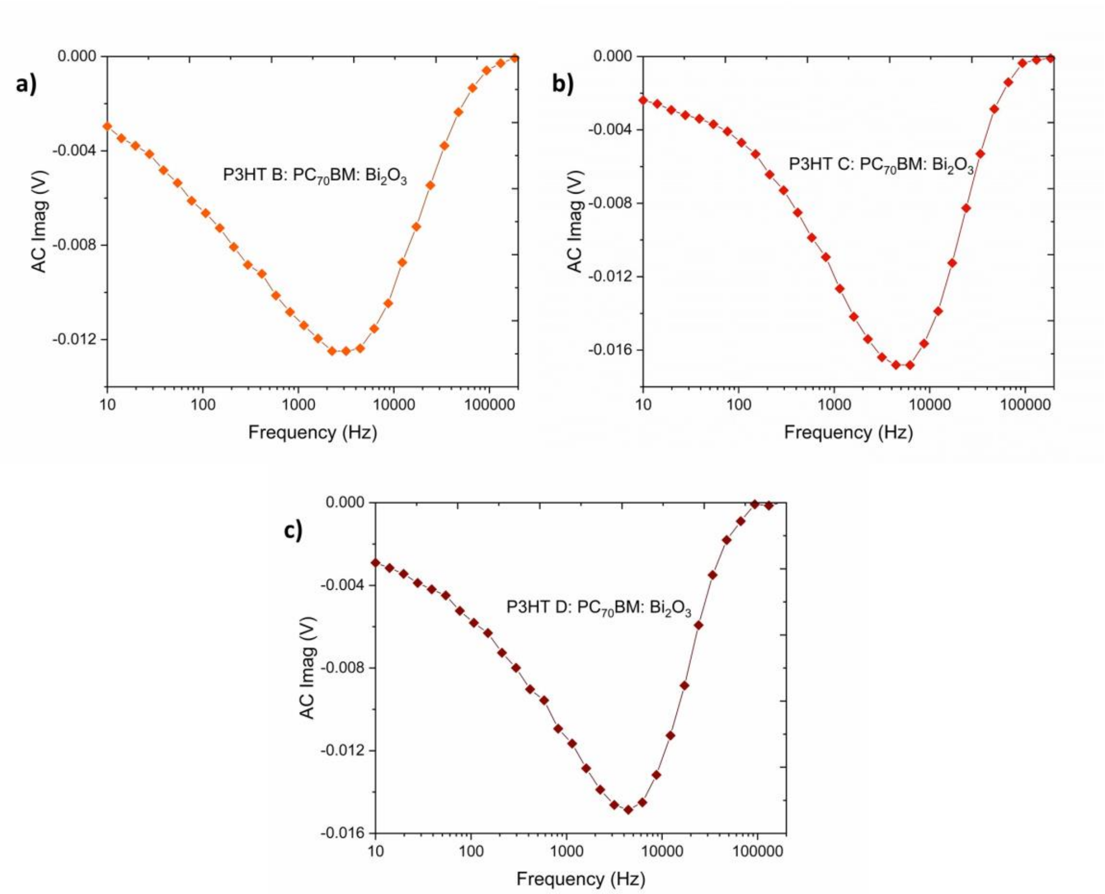

Figure S7. IMVS spectra of the NP-BHJ detectors fabricated with a) P3HT B, b) P3HT C, c) P3HT D.

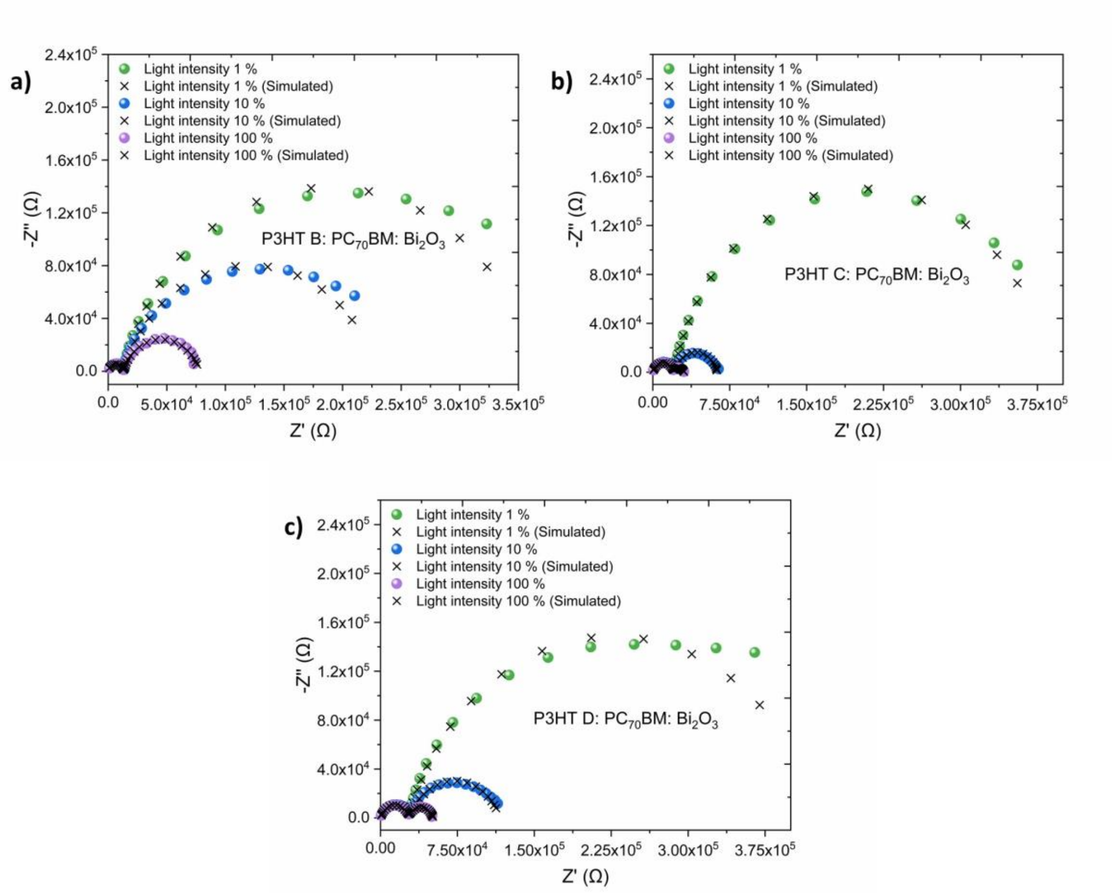

Figure S8. Nyquist plots of the NP-BHJ detector fabricated with a) P3HT B, b) P3HT C, c) P3HT D under illumination light intensity conditions of 1, 10, and 100 % when biased at 0 V. The black colour crosses (×) represent the fits for each light intensity condition calculated using the equivalent circuit model.

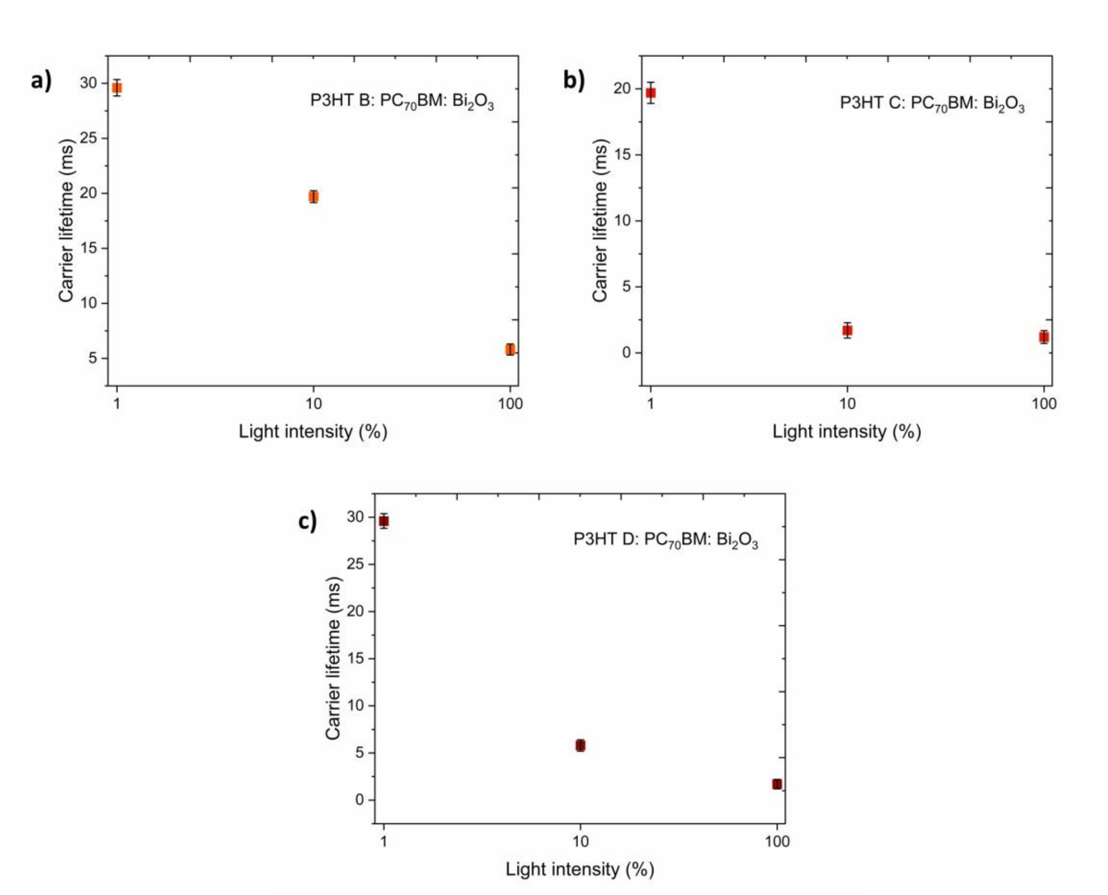

Figure S9. Carrier lifetime as a function of illumination light intensity of the NP-BHJ detectors fabricated with a) P3HT B, b) P3HT C, c) P3HT D.

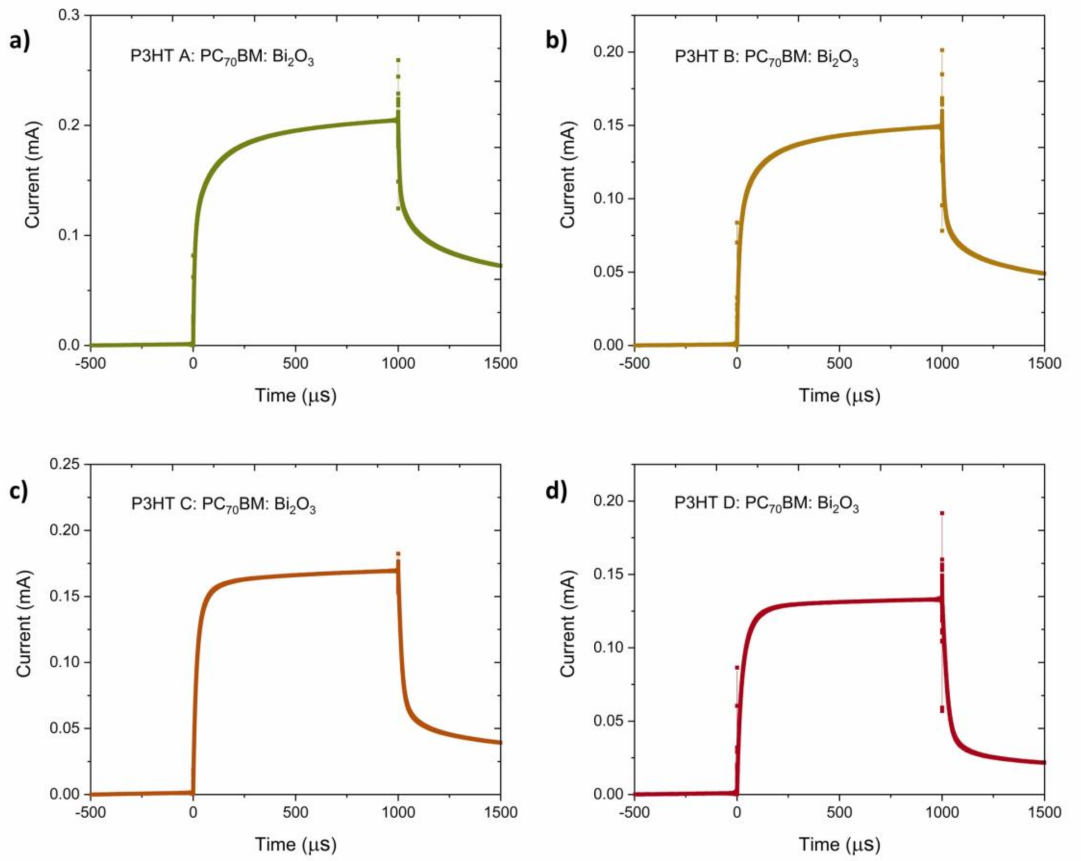

Figure S10. Photocurrent transient of the NP-BHJ detectors fabricated with a) P3HT A, b) P3HT B, c) P3HT C, d) P3HT D obtained from TPC under an applied bias of -10 V.

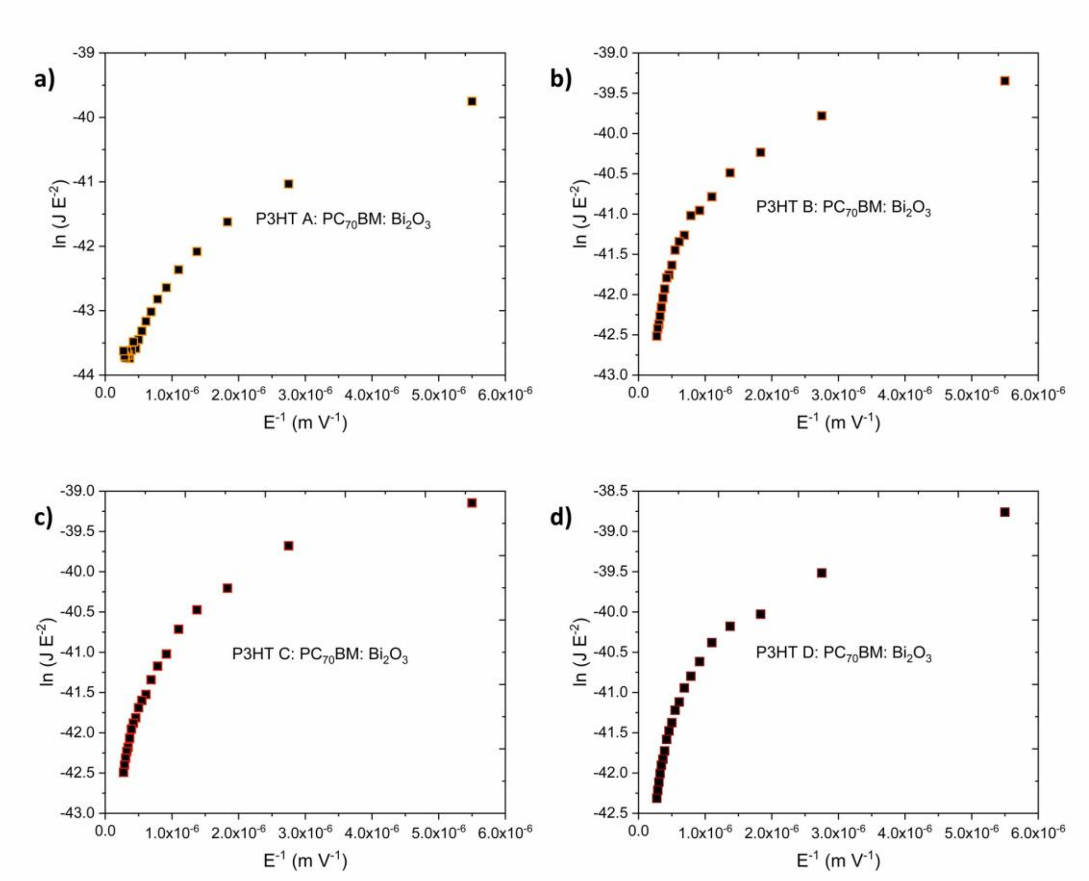

Figure S11. $ln(JE^{-2})$ vs E^{-1} plots for the dark diode characteristics of the NP-BHJ detectors fabricated with a) P3HT A, b) P3HT B, c) P3HT C, d) P3HT D under reverse bias.

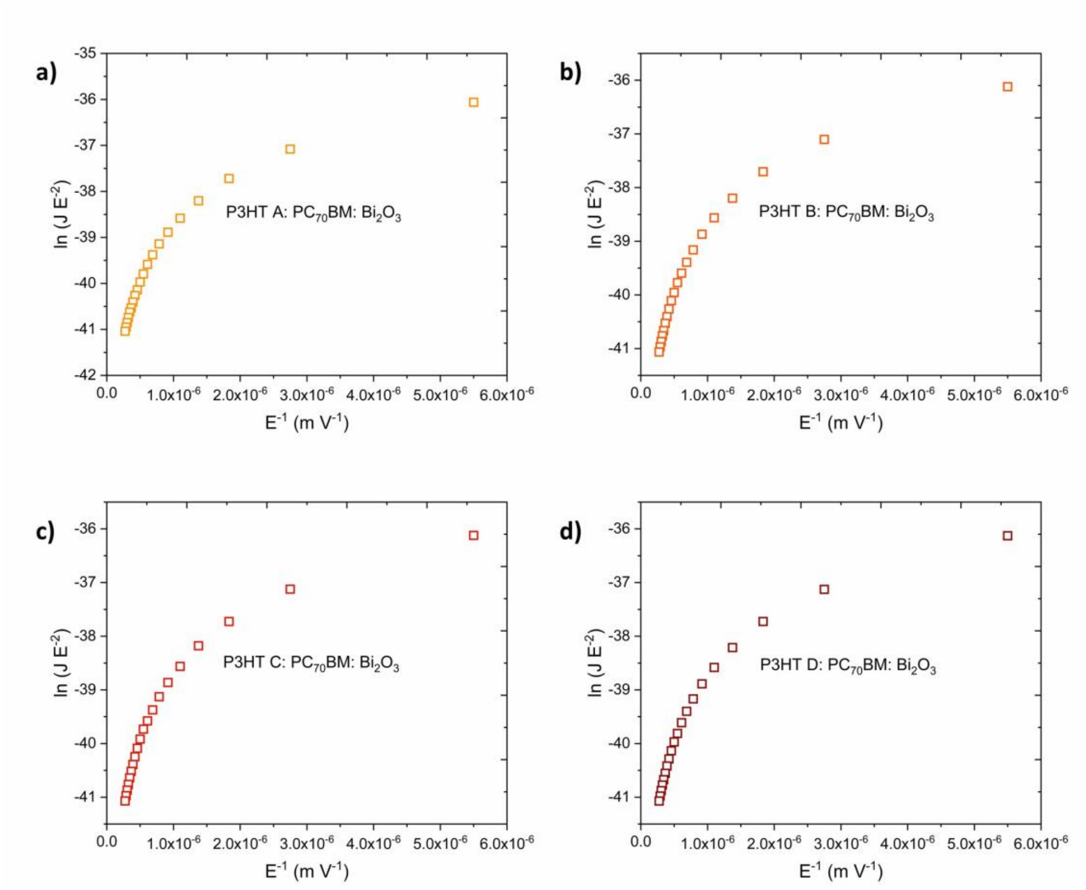

Figure S12. $ln(JE^{-2})$ vs E^{-1} plots for the X-ray photocurrent response characteristics of the NP-BHJ detectors fabricated with a) P3HT A, b) P3HT B, c) P3HT C, d) P3HT D under reverse bias.

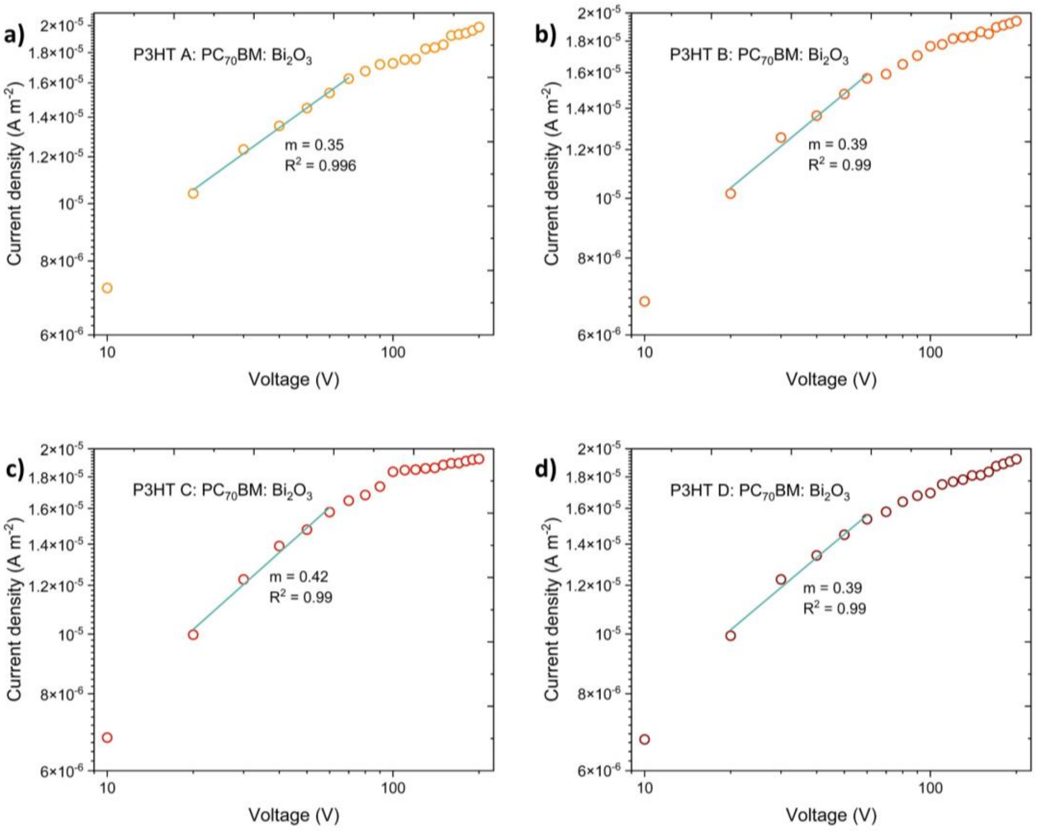

Figure S13. Current density-voltage plots for the X-ray photocurrent response characteristics of the NP-BHJ detectors fabricated with a) P3HT A, b) P3HT B, c) P3HT C, d) P3HT D under reverse bias. Extracted gradient (m) and the linear regression (R^2) are given next to each linear fit.

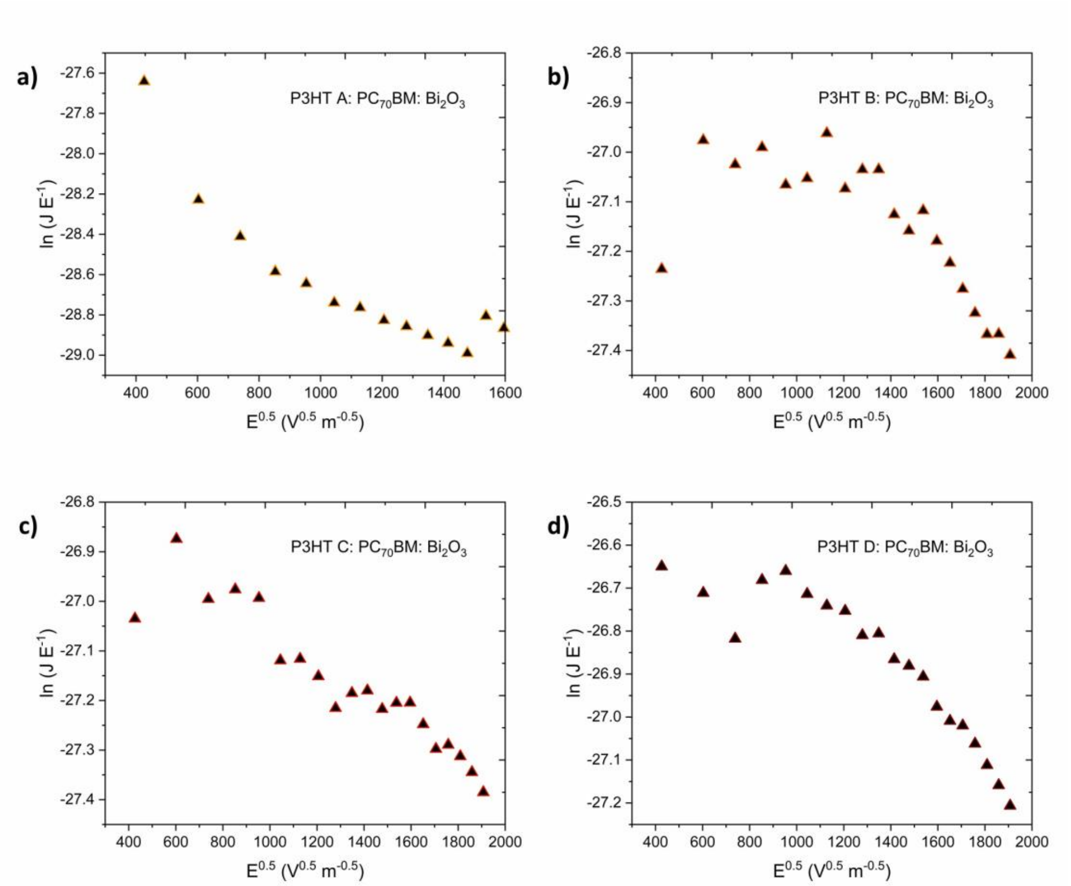

Figure S14. $ln(JE^{-1})$ vs $E^{0.5}$ plots for the dark diode characteristics of the NP-BHJ detectors fabricated with a) P3HT A, b) P3HT B, c) P3HT C, d) P3HT D under reverse bias.

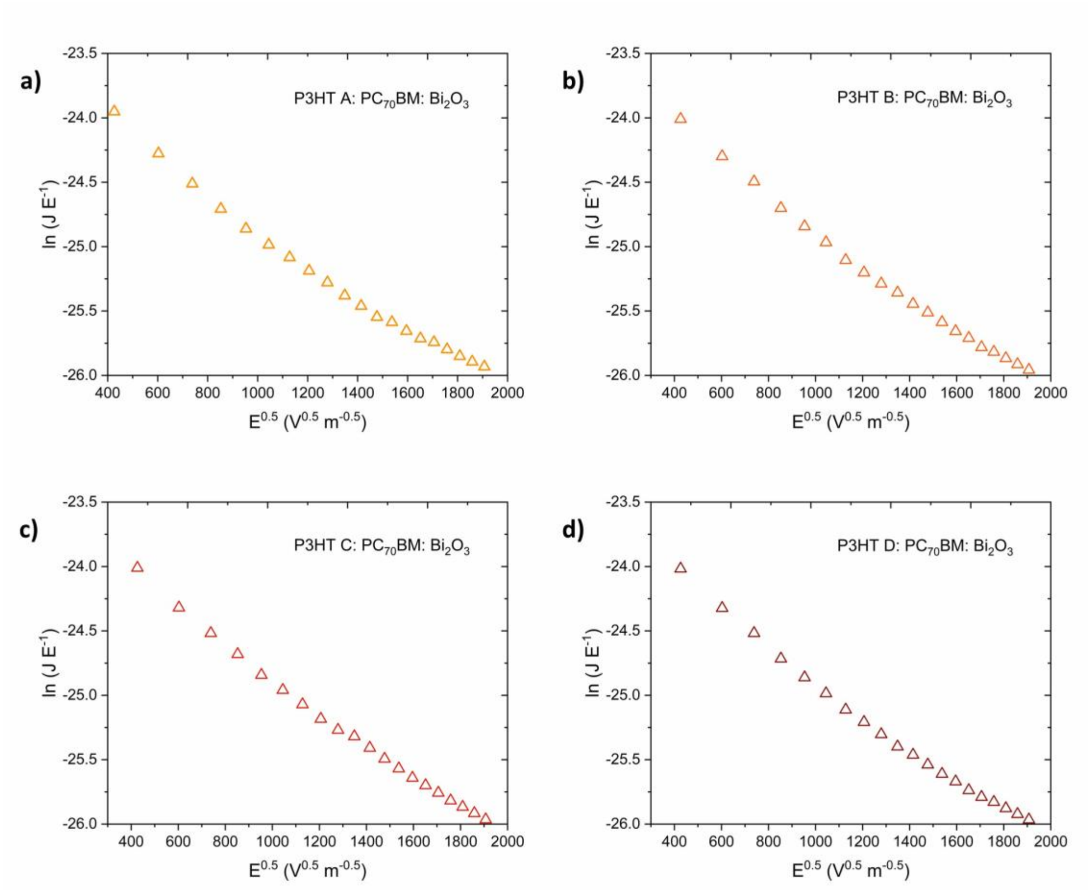

Figure S15. $ln(JE^{-1})$ vs $E^{0.5}$ plots for the X-ray photocurrent response characteristics of the NP-BHJ detectors fabricated with a) P3HT A, b) P3HT B, c) P3HT C, d) P3HT D under reverse bias.

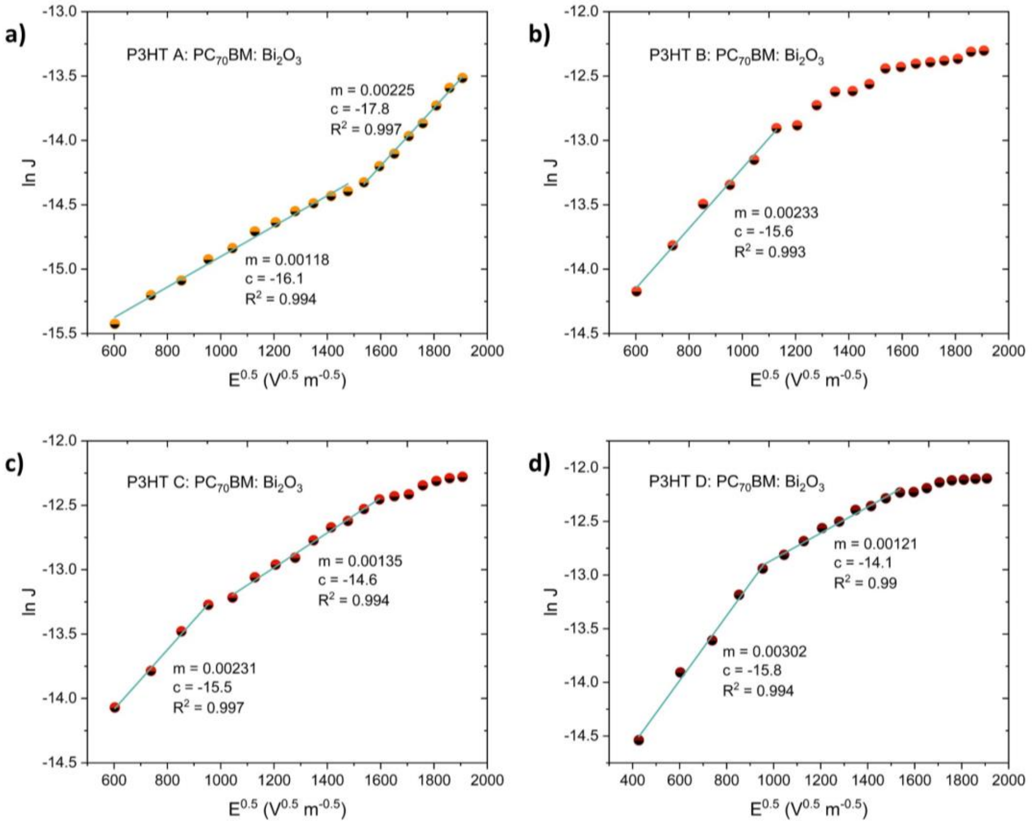

Figure S16. $ln(J)$ vs $E^{0.5}$ plots for the dark diode characteristics of the NP-BHJ detectors fabricated with a) P3HT A, b) P3HT B, c) P3HT C, d) P3HT D under reverse bias. Extracted gradient (m), intercept (c), and the linear regression (R^2) are given next to each linear fit.

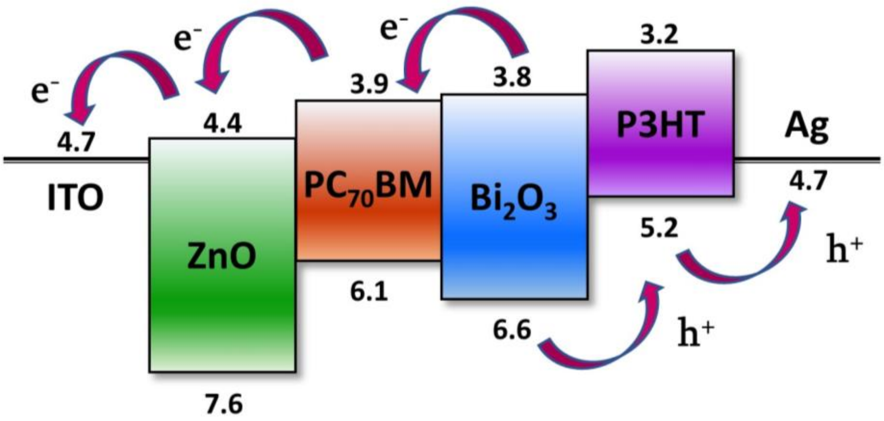

Figure S17. Flat band diagram for the NP-BHJ X-ray detectors indicating the energy band gap between Bi_2O_3, P3HT, and $PC_{70}BM$.

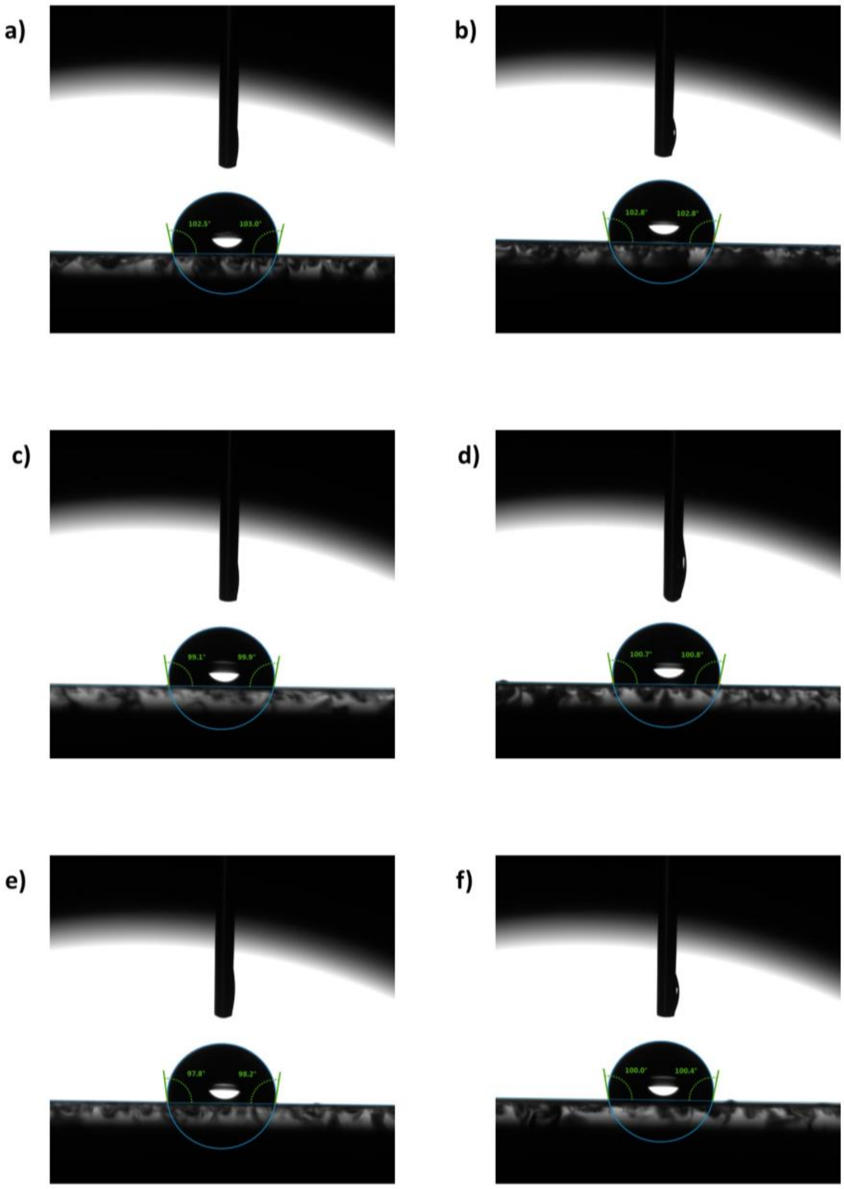

Figure S18. Surface free energy measurement of the a) P3HT B film, b) P3HT B: $PC_{70}BM$: Bi_2O_3 blend film, c) P3HT C film, d) P3HT C: $PC_{70}BM$: Bi_2O_3 blend film, e) P3HT D film, f) P3HT D: $PC_{70}BM$: Bi_2O_3 blend film using the sessile drop contact angle method.

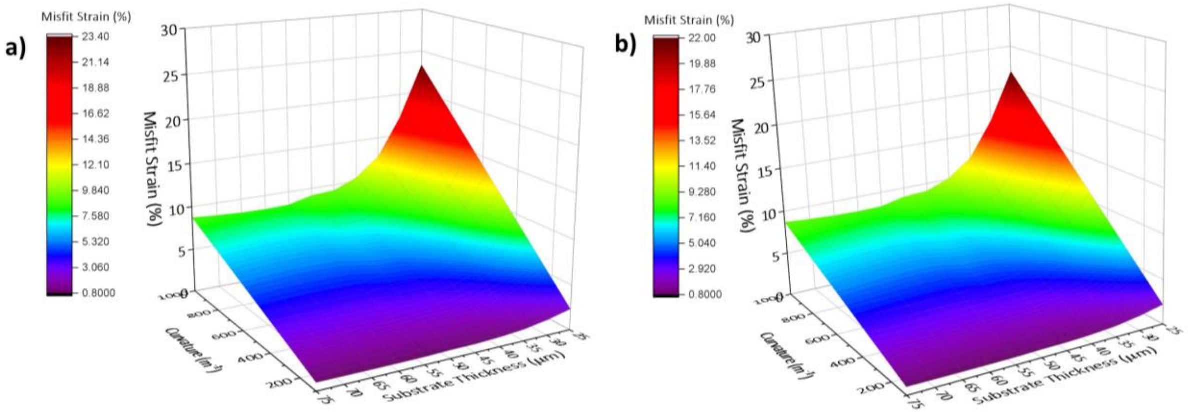

Figure S19. Misfit strain as a function of both radius of curvature and substrate thickness for NP-BHJ film based on a) P3HT B and b) P3HT C.

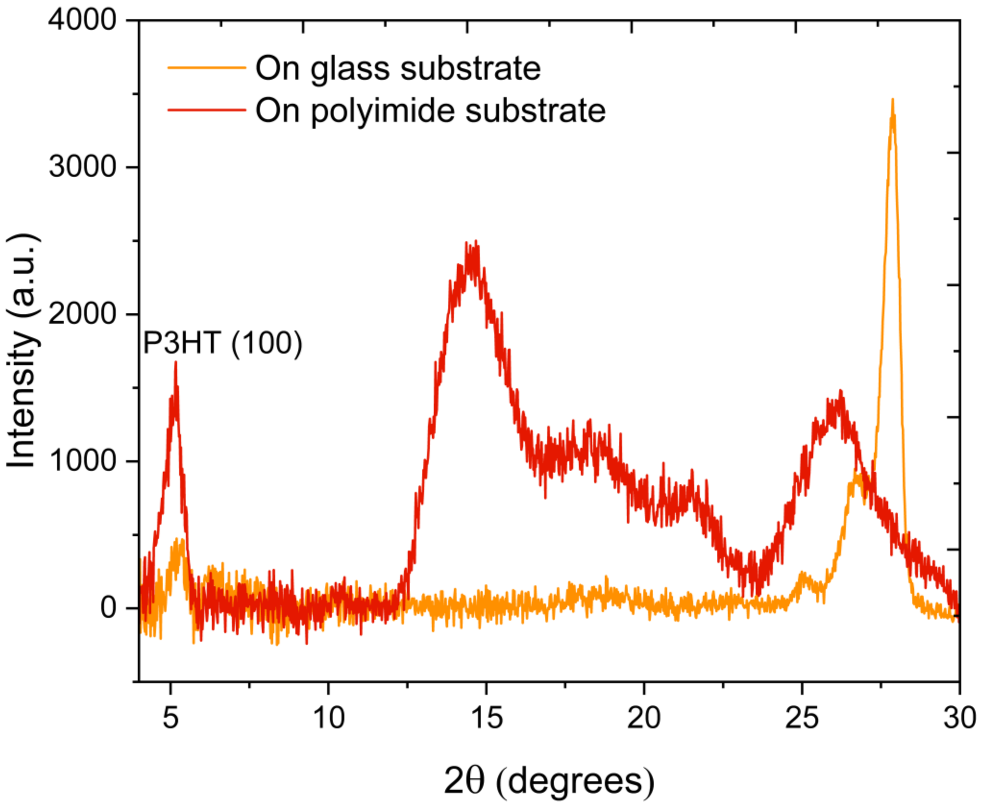

Figure S20. Comparison of 1D line profiles of the NP-BHJ film based on P3HT D fabricated on 75 μm thick polyimide substrate and glass substrate.

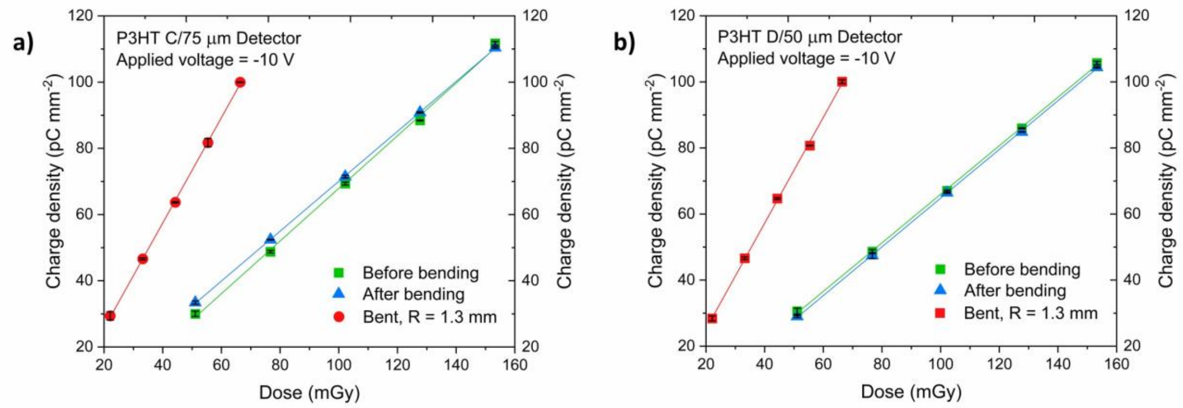

Figure S21. Charge density as a function of the incident dose for the a) P3HT C/75 μm and b) P3HT D/50 μm detector measured before bending (green solid squares), during bending with a bending radius of 1.3 mm (red solid circles), and after bending (blue solid triangles) indicating dose linearity under each condition.

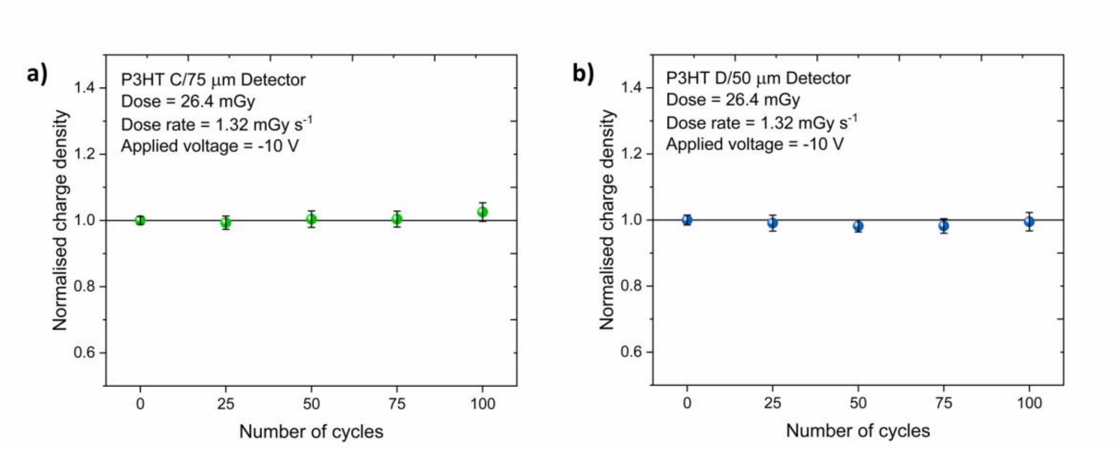

Figure S22. Normalised charge density of the a) P3HT C/75 μm and b) P3HT D/50 μm detector measured before bending, and after 25, 50, 75, and 100 bending cycles.

[1] K. Itonaga, T. Arimura, K. Matsumoto, G. Kondo, K. Terahata, S. Makimoto, M. Baba, Y. Honda, S. Bori, T. Kai, K. Kasahara, M. Nagano, M. Kimura, Y. Kinoshita, E. Kishida, T. Baba, S. Baba, Y. Nomura, N. Tanabe, N. Kimizuka, Y. Matoba, T. Takachi, E. Takagi, T. Haruta, N. Ikebe, K. Matsuda, T. Niimi, T. Ezaki, T. Hirayama, in *Dig. Tech. Pap. - Symp. VLSI Technol.*, Institute Of Electrical And Electronics Engineers Inc., **2014**.

[2] C. Choi, M. K. Choi, S. Liu, M. S. Kim, O. K. Park, C. Im, J. Kim, X. Qin, G. J. Lee, K. W. Cho, M. Kim, E. Joh, J. Lee, D. Son, S. H. Kwon, N. L. Jeon, Y. M. Song, N. Lu, D. H. Kim, *Nat. Commun.* **2017**, *8*, 1.

[3] B. Guenter, N. Joshi, R. Stoakley, A. Keefe, K. Geary, R. Freeman, J. Hundley, P. Patterson, D. Hammon, G. Herrera, E. Sherman, A. Nowak, R. Schubert, P. Brewer, L. Yang, R. Mott, G. Mcknight, *Opt. Express.* **2017**, *25*, 12.

[4] C. Choi, J. Leem, M. S. Kim, A. Taqieddin, C. Cho, K. W. Cho, G. J. Lee, H. Seung, H. J. Bae, Y. M. Song, T. Hyeon, N. R. Aluru, S. W. Nam, D. H. Kim, *Nat. Commun.* **2020**, *11*, 1.

[5] Y. Sun, J. A. Rogers, *Adv. Mater.* **2007**, *19*, 1897.

[6] D. Y. Khang, H. Jiang, Y. Huang, J. A. Rogers, *Science.* **2006**, *311*, 208.

[7] D. H. Kim, J. H. Ahn, M. C. Won, H. S. Kim, T. H. Kim, J. Song, Y. Y. Huang, Z. Liu, C. Lu, J. A. Rogers, *Science.* **2008**, *320*, 507.

[8] J. A. Rogers, in *TRANSDUCERS 2009 - 15th Int. Conf. Solid-State Sensors, Actuators Microsystems*, **2009**, pp. 1602–1603.

[9] J. A. Rogers, T. Someya, Y. Huang, *Science.* **2010**, *327*, 1603.

[10] S. Demchyshyn, M. Verdi, L. Basiricò, A. Ciavatti, B. Hailegnaw, D. Cavalcoli, M. Clark Scharber, N. Serdar Sariciftci, M. Kaltenbrunner, B. Fraboni, S. Demchyshyn, B. Hailegnaw, M. Kaltenbrunner, M. Verdi, L. Basiricò, A. Ciavatti, D. Cavalcoli, B. Fraboni, M. C. Scharber, N. S. Sariciftci, *Adv. Sci.* **2020**, *7*, 24.

[11] A. J. J. M. van Breemen, M. Simon, O. Tousignant, S. Shanmugam, J. L. van der Steen, H. B. Akkerman, A. Kronemeijer, W. Ruetten, R. Raaijmakers, L. Alving, J. Jacobs, P. E. Malinowski, F. De Roose, G. H. Gelinck, *npj Flex. Electron.* **2020**, *4*, 1.

[12] H. M. Thirimanne, K. D. G. I. Jayawardena, A. J. Parnell, R. M. I. Bandara, A. Karalasingam, S. Pani, J. E. Huerdler, D. G. Lidzey, S. F. Tedde, A. Nisbet, C. A. Mills, S. R. P. Silva, *Nat. Commun.* **2018**, *9*, 1.

[13] H. M. Thirimanne, K. D. G. I. Jayawardena, A. Nisbet, Y. Shen, R. M. I. Bandara, C. A. Mills, G. Shao, S. R. P. Silva, *IEEE Trans. Nucl. Sci.* **2020**, *67*, 2238.

[14] M. P. A. Nanayakkara, L. Matjačić, S. Wood, F. Richheimer, F. A. Castro, S. Jenatsch, S. Züfle, R. Kilbride, A. J. Parnell, M. G. Masteghin, H. M. Thirimanne, A. Nisbet, K. D. G. I. Jayawardena, S. R. P. Silva, *Adv. Funct. Mater.* **2020**, 2008482.

[15] P. A. Staniec, A. J. Parnell, A. D. F. Dunbar, H. Yi, A. J. Pearson, T. Wang, P. E. Hopkinson, C. Kinane, R. M. Dalgliesh, A. M. Donald, A. J. Ryan, A. Iraqi, R. A. L. Jones, D. G. Lidzey, *Adv. Energy Mater.* **2011**, *1*, 499.

[16] J. Kurpiers, D. Neher, *Nat. Publ. Gr.* **2016**, *6*, 1.

[17] R. J. Kline, M. D. McGehee, E. N. Kadnikova, J. Liu, J. M. J. Fréchet, *Adv. Mater.* **2003**, *15*, 1519.

[18] M. Jørgensen, K. Norrman, S. A. Gevorgyan, T. Tromholt, B. Andreasen, F. C. Krebs, *Adv. Mater.* **2012**, *24*, 580.

[19] N. R. Tummala, C. Risko, C. Bruner, R. H. Dauskardt, J.-L. Brédas, *J. Polym. Sci. Part B Polym. Phys.* **2015**, *53*, 934.

[20] L. Basiricò, A. Ciavatti, B. Fraboni, *Adv. Mater. Technol.* **2021**, *6*, 2000475.

[21] P. Büchele, M. Richter, S. F. Tedde, G. J. Matt, G. N. Ankah, R. Fischer, M. Biele, W. Metzger, S. Lilliu, O. Bikondoa, J. E. Macdonald, C. J. Brabec, T. Kraus, U. Lemmer, O. Schmidt, *Nat. Photonics* **2015**, *9*, 843.

[22] A. G. Dixon, R. Visvanathan, N. A. Clark, N. Stingelin, N. Kopidakis, S. E. Shaheen, *J. Polym. Sci. Part B Polym. Phys.* **2018**, *56*, 31.

[23] F. Liu, D. Chen, C. Wang, K. Luo, W. Gu, A. L. Briseno, J. W. P. Hsu, T. P. Russell, *ACS Appl. Mater. Interfaces* **2014**, *6*, 19876.

[24] F. A. Boroumand, M. Zhu, A. B. Dalton, J. L. Keddie, P. J. Sellin, J. J. Gutierrez, *Appl. Phys. Lett.* **2007**, *91*, 3.

[25] A. Intaniwet, J. L. Keddie, M. Shkunov, P. J. Sellin, *Org. Electron.* **2011**, *12*, 11.

[26] A. Ciavatti, E. Capria, A. Fraleoni-Morgera, G. Tromba, D. Dreossi, P. J. Sellin, P. Cosseddu, A. Bonfiglio, B. Fraboni, *Adv. Mater.* **2015**, *27*, 7213.

[27] L. Basirico, A. Ciavatti, M. Sibilia, A. Fraleoni-Morgera, S. Trabattoni, A. Sassella, B. Fraboni, *IEEE Trans. Nucl. Sci.* **2015**, *62*, 1791.

[28] G. H. Gelinck, A. Kumar, D. Moet, J. L. P. J. Van Der Steen, A. J. J. M. Van Breemen, S. Shanmugam, A. Langen, J. Gilot, P. Groen, R. Andriessen, M. Simon, W. Ruetten, A. U. Douglas, R. Raaijmakers, P. E. Malinowski, K. Myny, *IEEE Trans. Electron Devices* **2016**, *63*, 197.

[29] A. Intaniwet, C. A. Mills, M. Shkunov, P. J. Sellin, J. L. Keddie, *Nanotechnology* **2012**, *23*, 23.

[30] A. Ciavatti, T. Cramer, M. Carroli, L. Basiricò, R. Fuhrer, D. M. De Leeuw, B. Fraboni, *Appl. Phys. Lett.* **2017**, *111*, 183301.

[31] C. A. Mills, H. Al-Otaibi, A. Intaniwet, M. Shkunov, S. Pani, J. L. Keddie, P. J. Sellin, *J. Phys. D Appl. Phys.* **2013**, *46*, 27.

[32] Y. C. Kim, Y. S. Choi, S. Y. Lee, *Nature*. **2017**, *550*, 7674.

[33] S. Shrestha, R. Fischer, G. J. Matt, P. Feldner, T. Michel, A. Osvet, I. Levchuk, B. Merle, S. Golkar, H. Chen, S. F. Tedde, O. Schmidt, R. Hock, M. Rührig, M. Göken, W. Heiss, G. Anton, C. J. Brabec, *Nat. Photonics* **2017**, *11*, 436.

[34] W. Pan, H. Wu, J. Luo, Z. Deng, C. Ge, C. Chen, X. Jiang, W.-J. Yin, G. Niu, L. Zhu, L. Yin, Y. Zhou, Q. Xie, X. Ke, M. Sui, J. Tang, *Nat. Photonics* **2017**, *11*, 726.

[35] W. Wei, Y. Zhang, Q. Xu, H. Wei, Y. Fang, Q. Wang, Y. Deng, T. Li, A. Gruverman, L. Cao, J. Huang, *Nat. Photonics* **2017**, *11*, 315.

[36] Z. Gou, S. Huanglong, W. Ke, H. Sun, H. Tian, X. Gao, X. Zhu, D. Yang, P. Wangyang, *Phys. status solidi – Rapid Res. Lett.* **2019**, *13*, 1900094.

[37] H. Mescher, E. Hamann, U. Lemmer, *Sci. Rep.* **2019**, *9*, 1.

[38] K. D. G. I. Jayawardena, H. M. Thirimanne, S. F. Tedde, J. E. Huerdler, A. J. Parnell, R. M. I. Bandara, C. A. Mills, S. R. P. Silva, *ACS Nano* **2019**, *13*, 6973.

[39] M. Stephen, K. Genevičius, G. Juška, K. Arlauskas, R. C. Hiorns, *Polym. Int.* **2017**, *66*, 13.

[40] F. Laquai, D. Andrienko, C. Deibel, D. Neher, in *Adv. Polym. Sci.*, Springer New York LLC, **2017**, pp. 267–291.

[41] C. T. G. Smith, R. W. Rhodes, M. J. Beliatis, K. D. G. I. Jayawardena, L. J. Rozanski, C. A. Mills, S. R. P. Silva, *Appl. Phys. Lett.* **2014**, *105*, 073304.

[42] S. A. Moiz, I. A. Khan, W. A. Younis, K. S. Karimov, *Conducting Polymers*. **2016**, *5*, 91.

[43] S. R. P. Silva, J. D. Carey, R. U. A. Khan, E. G. Gerstner, J. V. Anguita, *Handb. Thin Film.* **2002**, 403.

[44] M. Kim, J. Lee, S. B. Jo, D. H. Sin, H. Ko, H. Lee, S. G. Lee, K. Cho, *J. Mater. Chem. A* **2016**, *4*, 15522.

[45] J. Yuan, T. Huang, P. Cheng, Y. Zou, H. Zhang, J. L. Yang, S. Y. Chang, Z. Zhang, W. Huang, R. Wang, D. Meng, F. Gao, Y. Yang, *Nat. Commun.* **2019**, *10*, 1.

[46] J. Als-Nielsen, D. Mcmorrow, *Elements of Modern X-Ray Physics Second Edition*, **2011**.

[47] M. Brinkmann, P. Rannou, *Adv. Funct. Mater.* **2007**, *17*, 101.

[48] M. Brinkmann, P. Rannou, *Macromolecules* **2009**, *42*, 1125.

[49] C. Bruner, R. Dauskardt, *Macromolecules* **2014**, *47*, 1117.

[50] S. Abbaszadeh, N. Allec, S. Ghanbarzadeh, U. Shafique, K. S. Karim, *IEEE Trans. Electron Devices* **2012**, *59*, 2383.

[51] S. Abbaszadeh, C. C. Scott, O. Bubon, A. Reznik, K. S. Karim, *Sci. Rep.* **2013**, *3*, 1.

[52] B. J. Briscoe, L. Fiori, E. Pelillo, *J. Phys. D. Appl. Phys.* **1998**, *31*, 2395.

[53] K. Zeng, Z. K. Chen, L. Shen, B. Liu, in *Thin Solid Films*, Elsevier, **2005**, pp. 111–118.

[54] W. C. Oliver, G. M. Pharr, *J. Mater. Res.* **1992**, *7*, 1564.

[55] L. Shen, L. Wang, T. Liu, C. He, *Macromol. Mater. Eng.* **2006**, *291*, 1358.

[56] H. C. Li, K. Koteswara Rao, J. Y. Jeng, Y. J. Hsiao, T. F. Guo, Y. R. Jeng, T. C. Wen, *Sol. Energy Mater. Sol. Cells* **2011**, *95*, 2976.

[57] J. Zang, F. Liu, *Appl. Phys. Lett.* **2008**, *92*, 21905.

[58] D. Bhatia, H. Sharma, R. S. Meena, V. R. Palkar, *Sens. Bio-Sensing Res.* **2016**, *9*, 45.

[59] J. S. Huang, C. Y. Chou, M. Y. Liu, K. H. Tsai, W. H. Lin, C. F. Lin, *Org. Electron.* **2009**, *10*, 1060.

[60] H. Looyenga, *Physica* **1965**, *31*, 401.

[61] B. Fraboni, A. Ciavatti, F. Merlo, L. Pasquini, A. Cavallini, A. Quaranta, A. Bonfi glio, A. Fraleoni-Morgera, B. Fraboni, A. Ciavatti, F. Merlo, L. Pasquini, A. Cavallini, A. Quaranta, A. Bonfi glio, A. Fraleoni-Morgera, *Adv. Mater* **2012**, *24*, 2289.

[62] B. Fraboni, A. Ciavatti, L. Basiricò, A. Fraleoni-Morgera, *Faraday Discuss.* **2014**, *174*, 219.

[63] G. Pipan, M. Bogar, A. Ciavatti, L. Basiricò, T. Cramer, B. Fraboni, A. Fraleoni-Morgera, *Adv. Mater. Interfaces* **2018**, *5*, 1700925.

[64] D. Zhao, M. Xu, B. Xiao, B. Zhang, L. Yan, G. Zeng, A. Dubois, P. Sellin, W. Jie, Y. Xu, *J. Mater. Chem. A* **2020**, *8*, 5217.

[65] H. Wei, H.-H. Fang, C. Wang, *Nat. Photonics* **2016**, *10*, 5.

[66] X. Wang, D. Zhao, Y. Qiu, Y. Huang, Y. Wu, G. Li, Q. Huang, Q. Khan, A. Nathan, W. Lei, J. Chen, *Phys. status solidi – Rapid Res. Lett.* **2018**, *12*, 1800380.

[67] J. A. Steele, W. Pan, C. Martin, M. Keshavarz, E. Debroye, H. Yuan, S. Banerjee, E. Fron, D. Jonckheere, C. W. Kim, W. Baekelant, G. Niu, J. Tang, J. Vanacken, M. Van der Auweraer, J. Hofkens, M. B. J. Roeffaers, *Adv. Mater.* **2018**, *30*, 1804450.

[68] H. Zhang, F. Wang, Y. Lu, Q. Sun, Y. Xu, B.-B. Zhang, W. Jie, M. G. Kanatzidis, *J. Mater. Chem. C* **2020**, *8*, 1248.

[69] C. Ji, S. Wang, Y. Wang, H. Chen, L. Li, Z. Sun, Y. Sui, S. Wang, J. Luo, *Adv. Funct. Mater.* **2020**, *30*, 1905529.

[70] Y. Zhang, Y. Liu, Z. Xu, H. Ye, Z. Yang, J. You, M. Liu, Y. He, M. G. Kanatzidis, S. (Frank) Liu, *Nat. Commun. 2020 111* **2020**, *11*, 1.

[71] A. Intaniwet, C. A. Mills, M. Shkunov, H. Thiem, J. L. Keddie, P. J. Sellin, *J. Appl. Phys.* **2009**, *106*, 6.

[72] G. N. Ankah, P. Büchele, K. Poulsen, T. Rauch, S. F. Tedde, C. Gimmler, O. Schmidt, T. Kraus, *Org. Electron.* **2016**, *33*, 201.

[73] L. Basiricò, A. Ciavatti, T. Cramer, P. Cosseddu, A. Bonfiglio, B. Fraboni, *Nat. Commun.* **2016**, *7*, 13063.

[74] H. M. Thirimanne, K. D. G. I. Jayawardena, A. J. Parnell, R. M. I. Bandara, A. Karalasingam, S. Pani, J. E. Huerdler, D. G. Lidzey, S. F. Tedde, A. Nisbet, C. A. Mills, & S. R. P. Silva, *Nat. Commun.* **2018**, *9*, 1.

[75] L. Basiricò, A. Ciavatti, I. Fratelli, D. Dreossi, G. Tromba, S. Lai, P. Cosseddu, A. Bonfiglio, F. Mariotti, C. Dalla Val, V. Bellucci, J. E. Anthony, B. Fraboni, *Front. Phys.* **2020**, *8*, 13.

[76] S. Yakunin, M. Sytnyk, D. Kriegner, S. Shrestha, M. Richter, G. J. Matt, H. Azimi, C. J. Brabec, J. Stangl, M. V Kovalenko, W. Heiss, *Nat. Photonics* **2015**, *9*, 7.

[77] L. Basiricò, S. P. Senanayak, A. Ciavatti, M. Abdi-Jalebi, B. Fraboni, H. Sirringhaus, *Adv. Funct. Mater.* **2019**, *29*, 1902346.

[78] W. Pan, B. Yang, G. Niu, K.-H. Xue, X. Du, L. Yin, M. Zhang, H. Wu, X.-S. Miao, J. Tang, *Adv. Mater.* **2019**, *31*, 1904405.

[79] Y. Xu, B. Jiao, T.-B. Song, C. C. Stoumpos, Y. He, I. Hadar, W. Lin, W. Jie, M. G. Kanatzidis, *ACS Photonics* **2018**, *6*, 196.

[80] G. J. Matt, I. Levchuk, J. Knüttel, J. Dallmann, A. Osvet, M. Sytnyk, X. Tang, J. Elia, R. Hock, W. Heiss, C. J. Brabec, *Adv. Mater. Interfaces* **2020**, *7*, 1901575.

[81] H. Mescher, F. Schackmar, H. Eggers, T. Abzieher, M. Zuber, E. Hamann, T. Baumbach, B. S. Richards, G. Hernandez-Sosa, U. W. Paetzold, U. Lemmer, *ACS Appl. Mater. Interfaces* **2020**, *12*, 15774.

[82] M. Hu, S. Jia, Y. Liu, J. Cui, Y. Zhang, H. Su, S. Cao, L. Mo, D. Chu, G. Zhao, K. Zhao, Z. Yang, S. F. Liu, *ACS Appl. Mater. Interfaces* **2020**, *12*, 16592.

[83] S. O. Kasap, J. A. Rowlands, *J. Mater. Sci. Mater. Electron.* **2000**, *11*, 179.

[84] J. H. Won, K. H. Kim, J. H. Suh, S. H. Cho, P. K. Cho, J. K. Hong, S. U. Kim, *Nucl. Instruments Methods Phys. Res. Sect. A Accel. Spectrometers, Detect. Assoc. Equip.* **2008**, *591*, 206.
